# Suppression of *Aedes* mosquito populations with the boosted sterile insect technique in tropical and Mediterranean urban areas

**DOI:** 10.1038/s41598-025-02795-1

**Published:** 2025-05-21

**Authors:** Jérémy Bouyer, David Almenar Gil, Ignacio Pla Mora, Vicente Dalmau Sorlí, Hamidou Maiga, Wadaka Mamai, Iris Claudel, Ronan Brouazin, Hanano Yamada, Louis-Clément Gouagna, Marie Rossignol, Fabrice Chandre, Marlène Dupraz, Frédéric Simard, Thierry Baldet, Renaud Lancelot

**Affiliations:** 1 ASTRE, CIRAD, INRAE, Univ. Montpellier, Montpellier, 34398 France; 2 ASTRE, CIRAD, INRAE, Univ. Montpellier, Plateforme Technologique CYROI, Sainte Clotilde, 97491 France; 3 Insect Pest Control Laboratory, Joint FAO IAEA Centre of Nuclear Techniques in Food and Agriculture, IAEA, Vienna, Austria; 4 Empresa de Transformación Agraria S.A., S.M.E, M.P., Paterna, Spain; 5 Servicio de Sanidad Vegetal, Generalitat Valenciana, Silla, Spain; 6 Direction Régionale Ouest, Institut de Recherche en Sciences de la Santé, Bobo-Dioulasso, Burkina Faso; 7 UMR MIVEGEC, Univ. Montpellier, IRD, CNRS, Saint-Pierre, La Réunion, 97410 France; 8 UMR MIVEGEC, Univ. Montpellier, IRD, CNRS, Montpellier, France

**Keywords:** Mosquito control, Vector control, Pyriproxyfen, Spain, La Reunion, Animal biotechnology, Genetic techniques, Epidemiology, Population dynamics, Invasive species

## Abstract

*Aedes* mosquitoes are the vectors of dengue viruses and other arboviruses, which threaten billions of people all over the world. The boosted sterile insect technique (boosted SIT) is a version of SIT in which irradiated sterile males also transmit a biocide to immature stages. We describe three field trials that were run in 2021: one against *Aedes aegypti* in La Reunion and two against *Aedes albopictus* in Spain, each using pyriproxyfen as a biocide. The relative density of adults (compared to their density in control sites: without sterile male release) decreased from 1.00 to 0.09, 95% credible interval [0.06, 0.15] (La Reunion, July) and to 0.02 [0.01, 0.03] and 0.11 [0.08, 0.16] (Spain, July and October). The success rate, corresponding to the proportion of traps with suppression greater than 80%, ranged from 0.43 to 0.71 in La Reunion, from 0.26 to 1.00, and from 0.50 to 0.70 in Spain. In Spain, suppression with boosted SIT was higher than with non-boosted SIT, in 2020 and 2022. This work is in line with the predictions of the model of a better efficacy of boosted SIT compared to SIT, together with partial protection from invasion of treated areas by fertile females, paving the way for larger-scale field trials.

## Introduction

*Aedes* mosquitoes, vectors of dengue viruses and other pathogens, are not efficiently controlled by conventional vector control tools: thus, new tools are needed. Recently, we proposed a new strategy, the boosted sterile insect technique (boosted SIT), whereby sterile males are used to disseminate biocides or biopesticides^1^. According to predictions from *a priori* models, this strategy, based on two complementary action modes, should be more efficient than SIT: on the one hand, sterility is induced in females mating with sterile males (SIT effect); on the other hand, the survival and molting rates of immature aquatic stages are affected by the biocide (boosted effect)^2–4^.

**Fig. 1:**
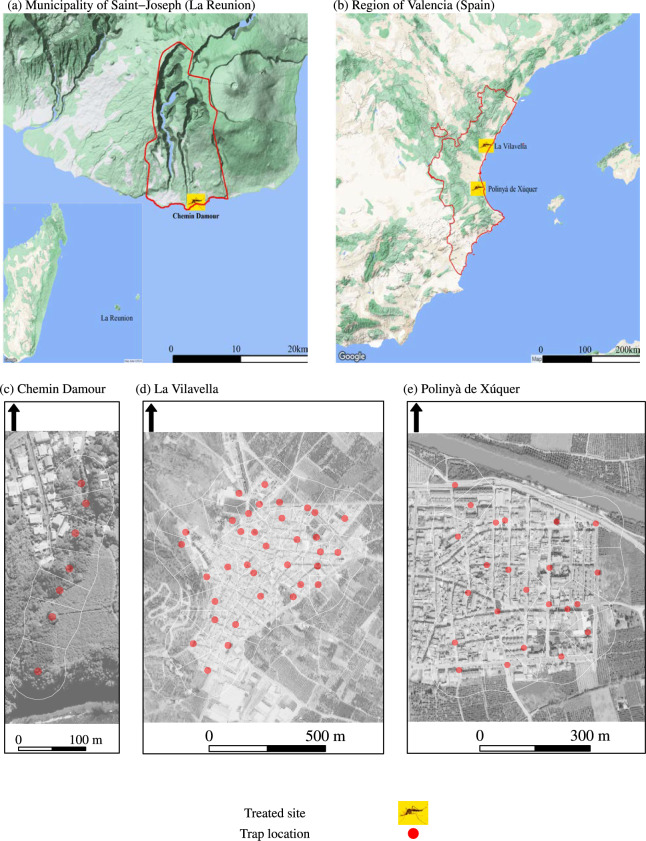
Sites selected for treatment with a pyriproxyfen-boosted sterile insect technique to suppress populations of *Aedes* mosquitoes. Trials were implemented from March to July 2021 in La Reunion (site 1) (graphs a, c), and from June to October 2021 in the Valencia Region, Spain (graphs b, d, e). The white-border polygons around the traps were defined by a Voronoi tessellation of trap locations. The background maps in graphs a, b, c, d and e were retrieved from the Google Maps Platform https://mapsplatform.google.com/, using functions available in the ggmap package for R https://github.com/features/packages version 4.0.0, together with a private API key. The administrative borders were retrieved from GADM https://gadm.org/data.html version 4.1.

**Table 1:** Main features of seven datasets fitted with a spatial Poisson model to assess the efficacy of boosted SIT during three field trials implemented in La Reunion (site 1) from March to July 2021, and in La Vilavella (site 2) and Polinyà de Xúquer (site 3) (Valencia Region, Spain) from June to October 2021.

Climate	Site	Region	Species	Stage	Zone	Treatment	Area (ha)	Releases/week/ha	Traps	Sessions	Counts
Tropical	1	La Reunion	*Aedes spp.*	Egg	Chemin Damour	Boosted SIT	10	353	7	63	1917
					Langevin	Control	5	0	5	45	2014
			*Aedes aegypti*	Adult	Chemin Damour	Boosted SIT	10	353	7	146	425
					Langevin	Control	5	0	5	82	856
			*Aedes albopictus*	Adult	Chemin Damour	Boosted SIT	10	353	7	146	333
					Langevin	Control	5	0	5	82	256
Mediterranean	2	Valencia	*Aedes albopictus*	Egg	La Vilavella	Boosted SIT	35	2712	35	630	16,514
					Betxí	Control	20	0	20	357	17,946
				Adult	La Vilavella	Boosted SIT	35	2712	35	630	1466
					Betxí	Control	20	0	20	357	2837
	3	Valencia	*Aedes albopictus*	Egg	Polinyà de Xúquer East	Boosted SIT	23	2620	23	392	8193
					Albalat de la Ribera	Control	59	0	59	991	43,319
				Adult	Polinyà de Xúquer East	Boosted SIT	23	2620	23	392	773
					Albalat de la Ribera	Control	59	0	59	991	7070

Each data set was the combination of two consecutive rows that described data (count of adults or eggs) from a treated zone and its associated control zone at a given site. The number of sessions was the product of the number of traps by the number of trapping dates. The counts corresponded to the total catches of adult mosquitoes or eggs throughout the study.

We report three coordinated field trials to assess the efficacy of pyriproxyfen-boosted SIT against *Aedes* mosquitoes in urban areas, both in tropical and Mediterranean environments (Table 1): La Reunion, site 1 (Fig. 1 graphs a and c) and Valencia, Spain (La Vilavella, site 2, Fig. 1, graphs b, d, and Polinyà de Xúquer, site 3, Fig. 1 graphs b and e). In each site, a zone treated with boosted SIT, that is, where boosted sterile males were released, was associated with a control zone, that is, without sterile male releases, and located in a neighboring area with a similar environment (SI Fig. S1)^5^.

Two *Aedes* vectors of dengue viruses are found in La Reunion^6,7^: *Aedes aegypti* (Linnaeus *in* Hasselquist, 1762) - the most important vector of dengue viruses worldwide^8,9^, and *Aedes albopictus* (Skuse, 1894), the main local vector of dengue viruses^10^. In a tropical environment such as La Reunion, *A. albopictus* shows a high ecological plasticity. It is ubiquitous up to an altitude of 1,200 m, in urban and rural environments. Its breeding sites are natural, as well as man-made, water collections, such as rock holes, saucers under flower pots, or larger rainwater tanks^11,12^. In contrast, *A. aegypti* is restricted to a few natural areas, in particular narrow valleys (*ravines*). In the drier West, it is developing in tree and rock holes^11^. In the wetter South, a hot spot for dengue virus transmission (Fig. 1 graph a), it is found in natural places with a high density of vacoa trees (Pandanaceae, *Pandanus utilis*) that was found to be its main larval habitat in Saint-Joseph^7^. This marginal distribution is attributed to competitive exclusion with *A. albopictus*, dominant in urban areas^13^. Competitive replacement, whereby a vector species can be favored by the reduction or elimination of another one, is well documented between *A. aegypti* and *A. albopictus*^14–16^. Several dengue epidemics occurred in La Reunion from 1977/1978 to 2021/2022^17–20^. The dengue virus is now considered endemic in this island^19,20^. *Aedes albopictus* is also the main vector of the chikungunya virus. From 2004 to 2006, a large outbreak was reported in La Reunion, with nearly 600,000 cases and more than 200 deaths^21–23^.

Mediterranean climatic conditions are met in the coastal Region of Valencia (Fig. 1 graph b), with mild winters and hot and dry summers. Human density is high, with villages and small cities scattered throughout a flood plain and croplands dominated by citrus. *Aedes albopictus* was first reported in 2013^24^. Then, it quickly spread, making it a nuisance with its aggressive diurnal feeding behavior. *Aedes albopictus* populations are not active all year long: data collected during a three-year monitoring survey revealed that the vector season spans from May to June to mid-October or November^25^. In addition, due to mass tourism and international trade, Valencia is at high risk of introduction of infections borne by *Aedes*, such as chikungunya, dengue, or Zika^24,26–29^. Finally, non-boosted SIT is routinely used with locally produced sterile males to control mosquitoes and Mediterranean fruit fly *Ceratitis capitata*, a major citrus pest in Valencia^25,30,31^.

We used a spatial Poisson model^32^ of mosquito relative density to evaluate the efficacy of boosted SIT in suppressing *Aedes* populations under the assumption that this efficacy results from the interaction of two effects: (i) the genetic effect of induced sterility in wild females and (ii) the biocidal effect of pyriproxyfen vectorized to breeding sites by boosted sterile males. The relative density was the density estimated from the traps located in the treated site, divided by the expected density, that is, the average density estimated from the traps located in the control site. Based on data collected during this field experiment, we expected that model predictions would show a decrease in the relative density of eggs or adult mosquitoes (primary outcome of the model), with a high probability of success (secondary outcome of the model). In this study, we did not evaluate the impact of treatment on dengue incidence because the study sites were not large enough (phase II trials^33^).

In La Reunion (site 1) (Fig. 1, graphs a, c), the field trial started in March 2021, that is, at the end of the hot and wet austral summer, with *Aedes* populations at the apex of their seasonal dynamics and the transmission risk of dengue viruses at its maximum^18,20^. Although the primary target was the homospecific *A. aegypti* population, we also evaluated the efficacy of boosted SIT in the heterospecific *A. albopictus* population (secondary target, by pyriproxyfen brought to *A. albopictus* larval sites by boosted sterile *A. aegypti*)). In Valencia (graphs b, d, e of Fig. 1), boosted sterile male *A. albopictus* were released before the start of the wild population to avoid a high density peak during the vector season. In addition, La Vilavella (site 2) was treated with non-boosted SIT in 2020 and 2022^25^. Furthermore, in 2021, Polinyà de Xúquer (site 3) was subdivided into two contiguous areas: Polinyà West, treated with non-boosted SIT, and Polinyà East, treated with boosted SIT. The mosquito control effort was described by the intensity of sterile male release (weekly number of sterile males/week/ha) and the ratio of sterile to wild males *s*/*w*.

The treated areas (Table 1) were subdivided into small polygons with the trap locations as their centroid, using Voronoi tessellation (Fig. 1). These polygons represent the spatial units of the relative density. A neighborhood matrix was built to estimate the spatial correlation between these units. This correlation structure was used to define and estimate the spatial random effect in the Bayesian spatial Poisson model of relative density^32^ (see Methods). The spatial random effect was divided into two components^34,35^:

- a white noise component, accounting for the fully random variations around the population mean (e.g., occurrence of highly-productive breeding sites such as drums in Spain, or rainwater tanks in La Reunion),
- a spatially structured component (conditional auto-regression model), accounting for spatial trends in the population mean (e.g., higher egg counts related to the immigration of gravid females).

This model was fitted for each treated site and mosquito stage, that is, a total of seven models (Table 1). Each of them had a single fixed effect: exposure to boosted SIT. With these settings, the fitted relative density was an estimate of the efficacy of boosted SIT in suppressing *Aedes* populations. The fitted models were used to build maps of the fitted relative density (the efficacy of the boosted SIT), as well as the probability of success of the boosted SIT (its statistical significance).

## Results

### Releases of sterile males

Boosted SIT was applied in a total area of 68 ha, with 5,671 inhabitants (Fig. 1): 10 ha in La Reunion (site 1) and 35 ha in La Vilavella (site 2) and 23 ha in Polinyà de Xúquer (site 3) (Valencia Region, Spain).

In La Reunion (site 1), a total of 59,972 sterile male *A. aegypti*, produced at the FAO-IAEA Insect Pest Control Laboratory in Vienna (Austria), were shipped by Fedex and treated locally with pyriproxyfen. They were released on nine occasions over a period of 17 weeks, from 15 March to 30 June 2021 (SI Fig. S2). The pace of the releases was irregular due to the reduced number of commercial flights between Vienna and La Reunion during the Covid-19 pandemics. In addition, transportation time often exceeded four days, the limit to keep sterile males alive^36^. The mean release density at the treated site was 353 sterile males boosted/ha/week. The estimates of the sterile to wild ratio for male *A. aegypti* were 0.1 for the first two months of the experiment, and 11.2 for the remaining time (mean: 0.7).

In Spain, all sterile males were reared in the Valencia medium-scale rearing facility^37^. They were released three times a week at each study site.

- In La Vilavella (site 2), a total of 3,892,075 boosted sterile male *A. albopictus* were released in 2021 over a 41 week period. The mean release density was 2712 (577) boosted sterile males/ha/week (standard deviation in brackets). The estimates of the monthly sterile-to-wild ratio for male *A. albopictus* were 64.2, 26.4, 53.0, and 6.0 for months 1 to 4 of the experiment (mean: 29.1). A total of 1887 boosted sterile males were collected in the release area, and only one in the Betxì control site, located 7.5 km from La Vilavella (site 2) (SI Fig. S1), thus showing a good isolation between the treated and control sites. This site was treated with non-boosted SIT in 2020 and 2022, with a release density of 2404 (839) and 1762 (516) untreated and unmarked sterile males/ha/week, respectively.
- In Polinyà de Xúquer (site 3), 2,470,465 boosted sterile males *A. albopictus* were released in the eastern part of this city and 2,418,350 untreated and unmarked sterile males in the western part in 2021, over a period of 41 weeks (SI Fig. S2). The mean release density was 2620 (487) boosted sterile males/ha/week in Polinyà East and 2681 (457) sterile males/ha/week in Polinyà West, from 15 June to 15 October 2021. The sterile-to-wild ratios for males could not be estimated because the non-boosted SIT and boosted SIT sites were contiguous, and unmarked sterile males could not be distinguished from wild males. In fact, 450 boosted sterile males were caught in the western area (treated with non-boosted SIT), that is, 16.7% of the total recaptured boosted sterile males, while 2225 were caught in the eastern area (treated with boosted SIT). Due to this high spread rate of boosted sterile males from Polinyà East to Polinyà West, and because unmarked (non-boosted) sterile males could not be distinguished from wild males (thus resulting in an unknown spread rate of sterile males from Polinyà west to Polinyà east), any relative density comparison between these two sites would be biased, to an unknown extent. Therefore, we collectively decided not to compare non-boosted SIT with boosted SIT in Polinyà East and Polinyà West in 2021. In addition, 14 boosted sterile males were caught at the control site, Albalat de la Ribera, located more than 1 km from the release area (SI Fig. S1). This was evidence that Albalat de la Ribera was not fully isolated from Polinyà de Xúquer (site 3). However, because this mosquito flow between the treated and control sites was quite limited, we validated the use of Albalat de la Ribera as the control site for Polinyà East.

### Efficacy of boosted SIT

To standardize the observed density (egg or adult counts), the monitoring period was cut into time intervals of approximately one month (see “Methods” section). For a given month, the relative density observed in the treated site was defined as the observed density divided by the expected density during the same month, that is, the average trap level density recorded in the control site. Therefore, time trends other than the effect of boosted SIT on population dynamics were removed, such as the influence of heavy rainfall. For each trial, the control and treated sites were selected in similar and neighboring environments (SI Fig. S1).

**Fig. 2:**
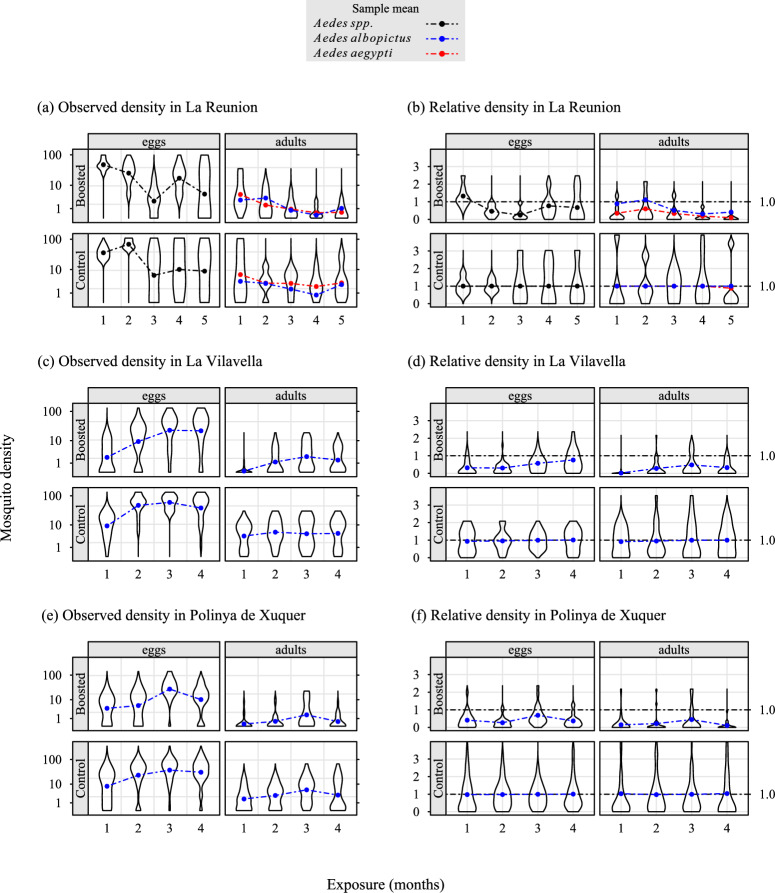
Observed density (graphs a, c, e), and observed relative density (graphs b, d, f) of *Aedes* mosquitoes ($\log _{10}$ scale) sampled during three boosted SIT trials in La Reunion (site 1) (treated site $n=7$ traps/collection day, vs. control site $n=5$), from March to July 2021 (La Reunion (site 1): graphs a, b), La Vilavella (site 2) ($n=35$ vs. $n=20$) (Spain: graphs c, d), and Polinyà de Xúquer (site 3) ($n=23$ vs. $n=55$) (Spain: graphs e, f), from June to October 2021. Eggs were a mixture of *Aedes albopictus* and *Aedes aegypti* in La Reunion, and *Aedes albopictus* alone in Spain. The observed relative density was the observed density in traps located in the treated site, divided by the expected density, i.e., the average density in the control site. For adults, the violin plots represented the probability density of *Aedes aegypti* in La Reunion, and *Aedes albopictus* in Spain. Eggs were sampled with oviposition traps. In La Reunion (site 1), adults were sampled with BG-Sentinel traps; in Spain, they emerged from the eggs collected with oviposition traps. In each site, the same number of traps were used for eggs and adults.

In this context, the relative density was an estimator of the efficacy of boosted SIT in suppressing mosquito populations. The observed density and the observed relative density are shown in Fig. 2, and the fitted relative densities in SI Table S1.

In La Reunion (site 1), 3931 *Aedes* eggs were collected from 12 oviposition traps, seven in the treated site and five in the control site (Table 1), from March to July 2021, during 108 trapping sessions. The total egg counts were analyzed because it was not possible to differentiate the two species (see “Methods” section). In addition, the hatching materials and the room were unfortunately contaminated with pyriproxyfen in the laboratory soon after the first release of sterile males treated with pyriproxyfen. Thus, it was not possible to compare the development rate from eggs to adults between the two sites (see “Methods” section).

A total of 1870 adults, that is, 1,281 *A. aegypti* and 589 *A. albopictus*, were sampled with 12 $\text {CO}_2$ baited BG-Sentinel traps (seven at the treated site and five at the control site), during 228 trapping sessions (Table 1). A density reduction was observed, much stronger for adults than for eggs. A break in sterile male releases, together with favorable conditions for the dynamics of the wild *Aedes* population, were associated with an increase in egg density at the treatment site during the second exposure month (Fig. 2 graph a), and a small relative density of adult *A. aegypti* at the treated site (Fig. 2 graph b). Subsequently, the pace of releases was more regular (SI Fig. S2). The lowest relative density of eggs was observed in the third month of exposure ($\theta =0.24$), then it increased: at the end of the trial, it was 0.67 (SI Table S1). In contrast, the rate of decay was roughly constant for the relative density of adult *A. aegypti*, reaching 0.09 (SI Table S1), i.e., an 11-fold reduction relative to the expected density. Although adult *A. albopictus* were not the primary targets of boosted SIT, their relative density decreased during the trial, reaching 0.41 at its end, i.e., a more than double reduction compared to the expected density of *A. albopictus*.

In Spain, a total of 85,972 eggs of *A. albopictus* were collected in 137 oviposition traps—35 and 23 in the treated sites of La Vilavella (site 2) and Polinyà de Xúquer (site 3), vs. 20 and 55 at the corresponding control sites (SI Fig. S1) during 2370 trapping sessions from June to October 2021. A total of 12,146 adults emerged from these eggs. In Betxì (control site for La Vilavella (site 2)), the apparent density of eggs *A. albopictus* increased strongly from June to July, then plateaued in August and September, before starting to decrease in October. The pattern was similar, but attenuated, in adult *A. albopictus*. The rate of development from eggs to adults was 59% in Betxì vs. 8% in La Vilavella (site 2) (treatment site). In La Vilavella (site 2), the apparent density of the eggs was on average 50% of the egg density in Betxì. It increased each month from June to October, but the density was always lower than in Betxì. In adults, the observed density peaked in September and decreased in October. The relative density (Fig. 2 graph d) was always below one in eggs and adults, that is, the mosquito density was always lower than in Betxì, whatever the stage of development and exposure. In eggs, the relative density increased from June to October. In adults, it peaked in September and decreased in October. In Albalat de la Ribera (control site for Polinyà de Xúquer (site 3) East), the observed density of eggs increased strongly from June to August, then decreased slightly afterward. The pattern was similar, but attenuated, in emerging adults. The development rates from eggs to adults were 63% in Albalat de la Ribera and 17% in Polinyà de Xúquer (site 3) East. In the latter treated site, the relative density of eggs (Fig. 2 graph f) was 44%. It increased during the first 3 months and decreased during the last one. In adults, the pattern was much attenuated with respect to eggs, with a peak in September. The rate of development from eggs to adults dropped to 4% in Polinyà de Xúquer (site 3) East, during the last month of exposure.

In all treated sites, the relative density (Fig. 2, SI Table S1) was most often below one in eggs and adults, that is, the mosquito density was lower than the expected density in the absence of treatment. There were two exceptions: eggs from *Aedes* and adult *A. albopictus*, both in La Reunion (site 1), that is, in heterospecific situations between the sterile male (*A. aegypti*) and its secondary target (*A. albopictus*).

### Non-boosted SIT vs. boosted SIT

Only egg data were available for comparisons. The average relative density of eggs of *A. albopictus* under non-boosted SIT (2020 and 2022), or boosted SIT (2021) in La Vilavella (site 2), as well as their two-way comparisons, are shown in Table 2 and SI Fig. S13. We used a permutation test to assess the statistical significance of the observed differences $\delta _o$ between the mean densities. To this end, 9999 random permutations of the treatment year were performed by blocks of trap $\times$ exposure month, thus ensuring the same design in each permuted data set as in the original. In La Vilavella (site 2), the efficacy in reducing egg density was higher with boosted SIT in 2021, than with non-boosted SIT in 2020 ($\delta _o=0.14$, $P=0.0066$) and with non-boosted SIT in 2022 ($\delta _o=0.26$, $P=0.0001$).

**Table 2:** Comparisons of average observed relative density of *A. albopictus* eggs sampled in La Vilavella (site 2) (Valencia, Spain) treated with non-boosted SIT (2020 and 2022), or boosted SIT (2021).

Difference terms					
1st	$\theta _1$	2nd	$\theta _2$	$\delta _o=\theta _1 - \theta _2$	$P(\ge \delta _o)$
Non-boosted SIT 20	0.63	Boosted 21	0.49	0.14	0.0081
Non-boosted SIT 22	0.75	Boosted 21	0.49	0.26	0.0001
Non-boosted SIT 22	0.75	Non-boosted SIT 20	0.63	0.13	0.0419

The observed relative density was the observed density in traps located in treated site ($n=35$ traps/collection day), divided by the expected density, i.e., the average observed density in the control site ($n=20$). A permutation test was used for these comparisons (9999 permutations), based on random permutations of the year among data blocks defined by the set of traps and each exposure month.

### Predictions of the spatial Poisson model

**Fig. 3:**
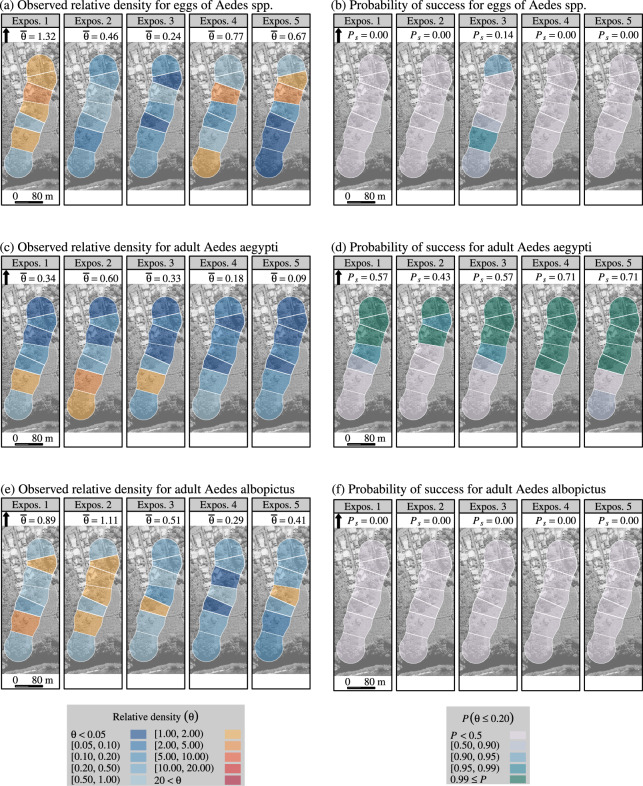
Efficacy of boosted SIT—with sterile male *Aedes aegypti*, in suppressing *Aedes* populations in La Reunion (site 1) during a field trial from March to July 2021. Relative density ($\theta$)—fitted with a spatial Poisson model, and probability of success ($P\left( \theta \le 0.20\right) \ge 0.95$) for *Aedes* eggs (graphs a, b), for adult *Aedes aegypti* (c, d), and for adult *Aedes albopictus* (graphs e, f). The observed relative density was the density observed in the treated site ($n=7$ traps/collection day), divided by the expected density, i.e., the average observed density in the control site ($n=5$). The probability of success was the proportion of traps in the treated site with a high probability ($P \ge 0.95$) that the actual relative density was 0.20 at the most.

**Fig. 4:**
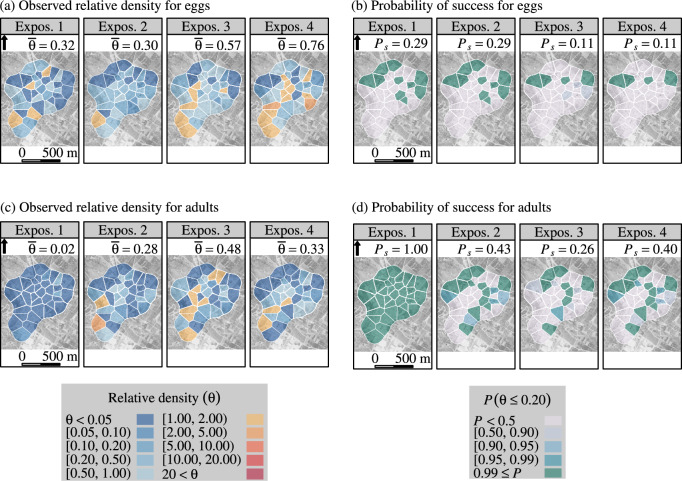
Efficacy of boosted SIT in suppressing *Aedes albopictus* population in La Vilavella (site 2) (Valencia, Spain) during a field trial from June to October 2021: relative density ($\theta$)— fitted with a spatial Poisson model and probability of success ($P\left( \theta \le 0.20 \right) \ge 0.95$) for *Aedes albopictus* eggs (graphs a, b), and adults (graphs c, d). The observed relative density were the density observed in traps located in the treated site ($n=35$ traps/collection day), divided by the expected density, i.e., the average observed density in the control site ($n=20$). The probability of success was the proportion of traps in La Vilavella (site 2) with a high probability ($P \ge 0.95$) that the actual relative density was 0.20 at the most.

**Fig. 5:**
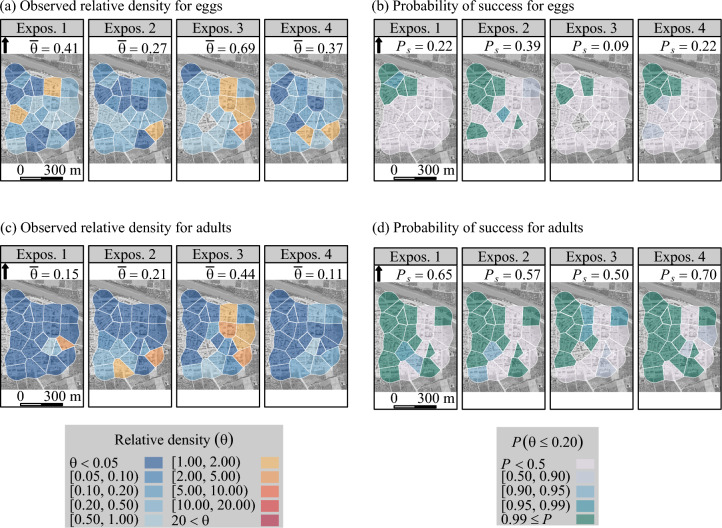
Efficacy of boosted SIT in suppressing *Aedes albopictus* population in Polinyà de Xúquer (site 3) East (Valencia, Spain) during a trial ranging from June to October 2021. Relative density ($\theta$)—fitted with a spatial Poisson model, and probability of success ($P\left( \theta \le 0.20\right) \ge 0.95$) for *Aedes albopictus* eggs (graphs a, b), and adults (graphs c, d). The observed relative density was the observed density in traps located in the treated site ($n=23$ traps/collection day), divided by the expected density, i.e., the average observed density in the control site ($n=55$). The probability of success was the proportion of traps in Polinyà de Xúquer (site 3) East with a high probability ($P \ge 0.95$) that the actual relative density was 0.20 at the most.

The success threshold for the boosted SIT was established at $\theta = 0.20$ (that is, 80% reduction in relative density, with respect to the expected density in the absence of treatment) for all treated sites and mosquito stages. We show the maps of trap-level relative density and success rate in Figs. 3, 4 and 5. Further insights on the fitted spatial Poisson models are provided in SI Figs. S6–S12.

The plot of time-space variations of relative density (Figs. 3, 4, 5) revealed a higher success rate for homospecific adults (primary target) than for eggs, suggesting that the latter were partially laid by immigrant females and did not emerge into adults under the effect of pyriproxyfen. This was obvious in La Reunion (site 1) (Fig. 3) where the efficacy of the boosted SIT was lower for *A. albopictus* than for *A. aegypti*. Consequently, the female *A. albopictus* contributed to increase the egg stock.

We did not observe any success in reducing the egg density in La Reunion (site 1) (Fig. 3), a likely consequence of permanent re-invasion by female *A. albopictus*. A strong reduction in egg density was observed, especially at the beginning of the trial (SI Fig. S6), but it was below the threshold of 80%. A high success rate was achieved for adult *A. aegypti* in the northern area (urban area). The relative density of the adult *A. albopictus* also decreased but did not reach the success threshold (Fig. 3). In any case (eggs, adult *A. aegypti* and *A. albopictus*), the random-effect maps did not highlight a meaningful spatial structure (SI Figs. S6–S8).

In La Vilavella (site 2), the reduction in adult density *A. albopictus* was stronger at the beginning of the trial than during the next three months, when the mosquito population increased (Fig. 2). The effect of boosted SIT was greater for eggs and adults in the northwest part of the site (Fig. 4), which leans against a mountainous area covered with xerophilic vegetation at the West (SI Fig. S1). Consistently, lower values of the structured random effect component were observed in the southern area of the site, in both adults and eggs (SI Figs. S9, 10).

In Polinyà de Xúquer (site 3), a strong reduction in egg density was observed. However, the success density threshold ($\theta = 0.20$) was only reached in the northwest part of the site. The success rate was higher in adults (Fig. 5). The north-western area was partially protected from *A. albopictus* re-invasion by a river in the north and by non-boosted SIT in the west. However, the eastern and southern parts of this site were surrounded by citrus plantations, where densities of *A. albopictus* are high^37^. The structured random effect component showed higher values in the eastern part than in the western part, for both eggs and adults (SI Figs. S11, 12).

## Discussion

### Efficacy of boosted SIT

The results of the data analysis are maps of the efficacy (fitted relative density) and the probability of success of the boosted SIT, that is, evidence of the possibility to suppress *Aedes* populations with the boosted SIT. From this perspective, it is a descriptive study of the successes and failures of boosted SIT. In the output maps, hot spots of relative density can help field experts identify highly productive breeding sites or areas. These maps can also be used to trigger ecological studies to formally identify breeding sites or other characteristics, as well as to adapt the releases of sterile males or the destruction of breeding sites by local mosquito control teams.

Boosted SIT suppressed *Aedes* populations in each treated site. In La Reunion (site 1), a strong efficacy was observed in adult *A. aegypti* (Fig. 3), although sterile males were released at a low rate and pace (SI Fig. S2). Furthermore, the trial started at the apex of seasonal population dynamics *Aedes*, the worst time for a non-boosted SIT program, that is, when it is the most difficult to reach a high ratio of sterile males to wild males^25^. This observation is in line with the predictions of a mathematical model developed to compare non-boosted SIT with boosted SIT: higher pyriproxyfen transport probably occurred when the female density was higher, although the induced sterility was comparatively lower^3,4^.

The efficacy of boosted SIT for *A. albopictus* was lower than for *A. aegypti*. In fact, the ratio of sterile to wild males was very low ($s/w=0.7$), compared to the recommended target of $s/w=10$ in the non-boosted SIT programs^5^, or to the achievement of $s/w=29.1$ in La Vilavella (site 2). Thus, in La Reunion (site 1), during the first two months of exposure, non-boosted SIT alone could not induce more than 10% sterility in female *A. aegypti*, assuming fully competitive sterile males. In non-boosted SIT trials, under these conditions, compensation for larval mortality would cancel any reduction in egg or adult density^38,39^. Therefore, most of the suppression was imputable to pyriproxyfen during this period.

The differential efficacy between *A. aegypti* and *A. albopictus* was also related to different larval habitats (container type and water volume) between the two species in La Reunion.

- *A. albopictus* larval habitats are mostly small containers (water volume < 1.5 L), including tree holes and plant leaves in ravines and plates in flower pots in houses. However, large containers (water volume ranging from 1.5 to 10 L) are also used as larval habitats by *A. albopictus*^7,12^, and may harbor large amounts of larvae.
- In contrast, at the treated site, larvae *A. aegypti* were only found in the canopy of vacoa trees, with low water volumes (less than 10 mL) in the stipes of the vacoa leaves^7^. Although *A. aegypti* can still be found in other habitats, mated females do not exploit the full range of *A. albopictus* larval habitats. In the areas of our study, they are not observed in large habitats.

In Spain, a moderate, although significant, incremental effect of the boosted SIT vs. non-boosted SIT was observed. This may be related to the predominant types of larval habitats of *A. albopictus* at the study sites, that is, mainly domestic drains, man-made ponds or pools without chlorine treatment, and drums (80% of the breeding sites), with larger water volumes than in La Reunion, thus reducing the boosting effect of pyriproxyfen^24,40^. Using boosted SIT in such settings would require the systematic removal of large breeding sites on private properties. Its feasibility and cost-effectiveness, as compared to non-boosted SIT, remain to be assessed. In particular, the low efficacy of the reported non-boosted La Vilavella (site 2) non-boosted SIT in 2022 (Table 2 and SI Fig. S13) was likely related to the up-scaling of non-boosted SIT in the Valencia Region, resulting in difficulties in reaching the targeted delivery of sterile males.

In La Reunion, the limited success of boosted SIT for *Aedes* eggs and adult *A. albopictus* was probably related to a strong immigration of adult female *A. albopictus* to the treated sites. In fact, the local population at this site was isolated from the other *A. aegypti* populations. In fact, the *A. aegypti* populations in La Reunion all belong to the same selvatic strain, which is only found in isolated natural locations (*ravines*), and not in urban environments^41^.

The situation is different for *A. albopictus*, which thrives in urban habitats^41^. The effect of pyriproxyfen on the fertility of wild females and the survival of immature stages still reduced the relative density of adult *A. albopictus* compared to the limited decrease in egg density. This protective effect against the immigration of fertile eggs is a great advantage of boosted SIT over non-boosted SIT. Consequently, in a reported non-boosted SIT trial in Greece^42^, an induced sterility of up to 50% in wild females did not cause any reduction in egg density. Furthermore, in our trials in Spain, better success rates were obtained for adults than for eggs, especially in traps located on the border of release areas (Figs 4, 5).

These trials showed the potential efficacy of boosted SIT for mosquito control. However, both non-boosted SIT and boosted SIT in a lower proportion showed high success rates only when they were implemented in isolated areas, or in areas large enough to mitigate the effect of active re-invasion by gravid females from neighboring untreated areas, as commonly observed in mosquito genetic control trials^25,43^. In general, suppression rates were higher than those reported in Kentucky during the first ten weeks after release^44^, with pyriproxyfen delivered by nonirradiated males. Using irradiated males for this purpose combined an induced sterility in wild females (the non-boosted SIT effect), with a lower molting rate and survival of immature stages (the biocide effect)^1^. The suppression rates were also higher than with induced sterility alone (non-boosted SIT effect) in Spain^25^. In La Reunion, a recent trial based on the release of up to 15,000 sterile males/ha/week led to a 50% induced sterility without significant suppression^45^. In neighboring Mauritius Island, the release of 16,667–20,000 sterile males/ha/week leads to 32% induced sterility concurrently with suppression 56%^46^. Our results are in line with those of a recent trial in China^47^. Boosting the Incompatible Insect Technique (IIT) with pyriproxyfen also resulted in a stronger efficacy to suppress mosquito populations. In addition, the authors reported that boosted IIT eliminated the high risk of population replacement when non-boosted IIT is used alone. The chronic toxicity of pyriproxyfen observed in adult males did not reduce their ability to induce cytoplasmic incompatibility. Likewise in our project, we did not observe any significant reduction of the flight ability in boosted males relative to non-boosted males (Dupraz et al. in prep). The formulation used in this study (40–60% mixture of pyriproxyfen and fluorescent dust designed to mark insects) has an impact on the quality of sterile males similar to fluorescent powder alone^48^.

This integrated strategy has two benefits:

- At low sterile-to-wild male ratios (leading to low induced sterility in wild females), pyriproxyfen still prevents the occurrence of compensation and overcompensation in larvae^38,39^, which is observed with non-boosted SIT, and does not allow the initial suppression of the target population. Compensation and over-compensation are related to density-dependent mortality of mosquito larvae. In fact, extrinsic larval mortality after mosquito control actions taken in an early larval stage can result in lower density-dependent larval mortality. In the end, the density of emerging adults may remain unchanged or even increase.
- When the ratio between sterile and wild males increases, the induced sterility in wild females becomes the main suppressor factor, thus reducing the risk of development of resistance to pyriproxyfen.

This strategy was previously proposed^49^ against insect pests. It is now widely used to control the Mediterranean fruit fly *Ceratitis capitata*^31,50,51^. Furthermore, as our results suggest, in a situation where both *A. albopictus* and *A. aegypti* are present, a cross effect (e.g., suppression of *A. aegypti* when the primary target is *A. albopictus*) could be expected, preventing re-invasion of an emptied ecological niche by the secondary target species^46,52^.

## Methods

### Efficacy and suppression

The definitions of efficacy and suppression were adapted from plant protection^53^: the efficacy of a treatment [against a pest population] is a defined, measurable and reproducible effect by a prescribed treatment; the suppression of a pest population is the application of a treatment in an infested area to reduce pest populations.

### Study sites

Three boosted SIT field trials were achieved in 2021 in (i) La Reunion: Chemin Damour (Saint-Joseph), 10 ha, Fig. 1 (graphs a and c), a small *ravine* (a narrow valley) terminated by the Indian Ocean at its southern end, and (ii) the Valencia Region (Spain): La Vilavella (site 2), 35 ha, Fig. 1 (graphs b and d), and eastern Polinyà de Xúquer (site 3), 23 ha, Fig. 1 (graphs b and e). A control site was associated with each treated site (SI Fig. S1): Langevin with Chemin Damour (La Reunion), Betxì with La Vilavella (site 2) and Albalat de la Ribera with Polinyà de Xúquer (site 3) (Valencia, Spain). In 2021, this latter site was subdivided into a western (22 ha) and an eastern (23 ha), respectively, treated with non-boosted SIT, and boosted SIT. In La Vilavella (site 2) and eastern Polinyà de Xúquer (site 3) boosted sterile male *A. albopictus* were released from February to November 2021.

### Production of the sterile males

The strain of *A. aegypti* used in La Reunion was colonized by the Institute of Research for Development (IRD) and ARS from local field collections. After several generations (more than ten), it was transferred to the Insect Pest Control Laboratory (IPCL) of the joint FAO/IAEA Center of Nuclear Sciences and Applications (Vienna, Austria). It was maintained in mass-rearing procedures developed at the IPCL^36^. The pupae were collected and separated by sex using mechanical and semi-automatic pupal sex sorters (John W. Hock Co., Gainesville, FL, USA; Wolbaki, Guangzhou, China). Emerging adult mosquitoes were maintained with a sucrose solution 10% until their irradiation. They were exposed to a 45 Gy dose using an X-ray blood irradiator (Raycell MK2)^54,55^. Sterile male mosquitoes were shipped to La Reunion using a long-distance transport protocol^36^.

In Spain, mosquitoes were reared and sterilized in the facilities of the non-boosted SIT project against *A. albopictus* from *Generalitat Valenciana*. The colony of *A. albopictus* was established in 2014 with eggs collected in various locations in the Valencia Region. Sterile males were produced under the conditions and protocol described in Tur *et al.*^25,37^. After irradiation, 750 pupae were allowed to emerge in plastic containers, featuring two sides of plastic mesh and with access to a sucrose solution 10%. On day of release, four to six days after irradiation, sterile male batches were separated from untreated mosquitoes kept for non-boosted SIT until release.

### Field and laboratory data

In La Reunion and Valencia, the *Aedes* populations were monitored using the same trap density of 1 trap/ha.

In La Reunion, traps were set to maximize the probability of detection of *A. aegypti*, as well as a balanced density ratio with *A. albopictus*^6,7^. The eggs were sampled with twelve oviposition traps every two weeks from February 03 to July 21, 2021, at the release and control sites (seven and five traps, respectively). The traps were 1000-mL black plastic pots filled with 250-mL of tap water. Two pieces of a green vacoa leaf were placed in each cup as an oviposition substrate. They were taken from the base of the leaf, and the spines were removed to provide a wider oviposition surface. The cups were placed in the leaves of a vacoa tree (or another tree when there was no vacoa around), 1.5–2 m from the ground, next to the trunk. They were collected after four days. Adults were also monitored using carbon dioxide-added carbon dioxide-added BG-Sentinel traps (200 mL/24 h), which were placed close to each oviposition cup.

The eggs were counted and dried in a greenhouse for 7 days ($27\,^{\circ }$C, 80% humidity). Then, the oviposition substrate was cut at the egg locations and placed for hatching in a 250 mL-Becher glass with 240 mL of water taken from the oviposition cups. Mosquito larvae were fed cat/rabbit food pellets daily for three days after eclosion. Larvae, pupae, and emerging adults were counted daily for 10 days. Preliminary data analysis revealed that the oviposition traps were unfortunately contaminated with pyriproxyfen in the laboratory soon after the first release of sterile males treated with pyriproxyfen. In fact, the rate of development of adult mosquitoes *Aedes* from eggs was 0.60 at the control site, during the week starting the day after the first release date. It dropped to 0.00 the following week. It increased again, up to 0.47 in early May, before falling to 0.00 from mid-May to the end of the trial.

In Spain, the *Aedes* monitoring network was made up of adult traps (Inestrap, Inesfly Corp. Paiporta, Spain) and oviposition traps. We only used data from oviposition traps to assess the efficacy of boosted SIT because the ovi-sticky traps had a capture ratio that was too low for a robust analysis. They were only used to assess the ratio of sterile to wild males. To this end, ten ovi-sticky traps (Inestrap) per block were checked weekly. The water deposit was filled with leftover, 100-$\upmu$m filtered, larval medium from the mass rearing facility. The sticky paper was replaced weekly and a cover paper was attached to preserve the samples. The captures were counted in the laboratory under a binocular microscope and later examined under a UV light source. They were classified into three categories: (i) female, (ii) marked male, and (iii) unmarked male. A total of 137 oviposition traps were installed at the control and boosted-SIT treated sites (Table 1). These traps were a 1 L black plastic container filled with tap water and a wooden slat. The slats were replaced weekly and transported to the laboratory in closed containers with dividers to avoid any contact between them. The water in the containers was filtered and stored in 1-L watertight glass jars. The slats were kept in a closed box in a room with 80% humidity and a temperature of $27\,^{\circ }$C for 3–4 days. *Aedes albopictus* eggs were then counted under stereo-microscope, separating complete and hatched eggs. One day later, the eggs were transferred to 500-mL plastic containers with watertight lids, filled up to 250 mL using the water collected from the same trap, possibly complete with tap water and 1 mL of hatching solution. The lid was closed and the container was kept at 25–27 $^\circ$C for 3 days. After that, the larvae were counted, adding 1 mL of the larval diet every 50 L1 larvae counted. Alive larvae were kept 15 days more. After this period, the adult *A. albopictus* emerged was counted.

### Treatment of sterile males with a formulation of pyriproxyfen

A powder mixture was prepared with 40% pyriproxyfen (Tagros, Chennai, India) and 60% fluorescein (pink fluorescent powder mix, DayGlo, Cleveland, USA). Plastic cups of 100-mL were coated with 1 mg of this formulation for each 100 adult males. These containers were taken to a cold room ($+4\,^{\circ }$C) for at least 30 min. Sterile males were then transferred to the cold room for immobilization for 5–10 min^56^. They were introduced into the containers which were rotated for 30 seconds (approximately 25 rotations).

### Release of sterile males

Due to restrictions for distant shipment, only small amounts of sterile male *A. aegypti* (up to 40,000 per week) were shipped from the FAO-IAEA Insect Pest Control Laboratory in Vienna, Austria, to La Reunion (10,442 km by plane, through Paris). In addition, a shipping break of 45 days occurred in March–April 2021 related to the Covid-19 pandemic. Six out of nine releases were achieved from the ground in five equally spaced fixed points (approximately 100 m) along *ravine*. The other releases were carried out by drone at an altitude of 50 m^33^.

In Spain, the releases began in February 2021, before the beginning of the *A. albopictus* reproductive season. Consequently, the mosquito breeding environment was likely contaminated with pyriproxyfen throughout the experiment. Because the effect of boosted SIT could only occur when the breeding activity of *A. albopictus* actually started, the starting date to assess the efficacy of mosquito control was established for June 15, 2021. Ground releases were achieved three times a week, at fixed points, with a density of one point/ha and one cage/point (approximately 750 males).

The intensity of releases was defined by:

- The weekly density of sterile males released treated with pyriproxyfen (mosquitoes/week/ha).
- The weekly sterile-to-wild male release ratio *s*/*w*, with *s*, the number of sterile males, and *w*, the number of homospecific wild males caught in the same traps during the same week. Data were grouped by longer periods to avoid zero counts in the denominator.

### Data analysis

#### Relative density and baseline surveys

Observed mosquito density, that is, trap-level mosquito count $y_i$, was standardized to relative density to eliminate the effect of population size and to remove any trend of relative density over time, other than exposure to boosted SIT, for example, the influence of meteorological variables on population dynamics. The expected density $E_i$ for exposure *i*, was the average of the observed densities at trap level at the control sites. The relative density $\theta _i$ for exposure *i* was the density observed at the trap level, divided by the expected density: $\theta _i = y_i / E_i$. Data were grouped by periods of approximately one month to avoid unstable estimates of relative density. The definition of periods was fine tuned to obtain balanced sub-sample sizes. Five periods alternating 31 and 30 days, starting on 4 March 2021, and four 34-day periods starting on 1st June 2021 were defined in La Reunion and Spain, respectively.

In La Reunion (site 1), the treated and control sites were selected after preliminary surveys^6,7^ to identify the breeding sites *Aedes* and optimize trap settings to provide unbiased estimates of each mosquito population. In addition, mosquito populations were monitored and compared at the control and treated sites, during 3 months before starting these releases (147 trapping sessions for adults, the same for eggs). We used a negative binomial model of the pre- and post-release density observed at the treated site for eggs *Aedes* or adults *A. albopictus*. Data were standardized with the expected density at the control site. The fixed part of the model had an intercept and a single fixed effect: the month of exposure to the boosted SIT, coded as a factor. Its reference level was 0, corresponding to pre-release observations. Thus, the exponentiated intercept was an estimate of the treated/control density ratio: 1.94 [1.28, 2.60] (95% CI) for adult *A. albopictus*, and 1.46 [1.04, 1.88] for eggs. This estimate was used as a multiplier of the expected density $E_i$.

In Valencia, *A. albopictus* populations were monitored in Polinyà de Xúquer (site 3) (treated site) and Albalat de La Ribera (control site) for one year before starting the field trial, which did not show significant differences in population dynamics^25^. Regarding La Vilavella (site 2), the Betxì control site could not be monitored before starting the field experiments. However, it was selected by an experimented team of professional field entomologists with a long history of mosquito monitoring surveys in the Valencia region.

#### The spatial Poisson model

The same statistical model was fitted to seven datasets defined by the combination of treated site, mosquito species, and development stage (Table 1). The modeling approach was taken from Moraga^32^. A mixed effect Poisson model of mosquito density was used to assess the efficacy of boosted SIT on *Aedes* density and its spatial variations. For the observed density $y_i~(i=1, \ldots ,I: \text {exposure month})$, the spatial Poisson model was as follows:

1 $$\begin{aligned} y_i \sim {\mathscr {P}}(E_i \times \theta _i), \end{aligned}$$

with $E_i$, and $\theta _i$ the expected and relative density. For the trap $j=1,\ldots , J$, the model was as follows:

2 $$\begin{aligned} \log (\theta _{i, j}) = \eta _{i,j} = \log (E_i) + b_1 + b_k\, x_k + u_j. \end{aligned}$$

- $\eta _{i,j}$ was the linear predictor for trap *j* at exposure *i*,
- $x_k$ ($k = 2, \ldots , I$) was an indicator variable taking the value 1 for exposure *k*, and 0 elsewhere,
- $b_1$ was the intercept i.e., the fixed-effect coefficient corresponding to the reference exposure $x_1$, and $b_k$ was the fixed-effect coefficient associated with $x_k$,
- $u_j$ was the trap-level realization of the spatial random effect *u*, with $\sum ^J_{j=1}u_j=0$, variance $\sigma ^2_u$.

With this parameterization, the relative density fitted for exposure *i* was an estimate of the effect of boosted SIT on relative density. It was the average of exponentiated linear predictors for the *J* traps at the treated site:

3 $$\begin{aligned} \theta _i = \frac{1}{J} \sum _{j=1}^J e^{\eta _{i, j}}. \end{aligned}$$

A modified version of the Besag–York–Mollié model^34,35^ was used for the variance of *u*, $\sigma ^2_u$. Thus, *u* was split into (i) a spatially structured component with a conditional auto-regressive (CAR) distribution, and (ii) a purely random, unstructured, “white noise” component.

The goodness of fit for these models is shown on SI Figs. S3 (La Reunion (site 1)), S4 (La Vilavella (site 2)) and S5 (Polinyà de Xúquer (site 3)), for eggs (left column), adult *A. albopictus* (right column) and adult *A. aegypti* (mid column, when present, that is, in la Reunion). The observed and fitted means were quite close. Plots of Pearson’s residuals did not reveal alarming trends.

The model coefficients were estimated in a Bayesian framework with integrated nested Laplace approximation (INLA): The Bayesian process is approximated by analytical solutions, allowing quick convergence^57^. The spatial Poisson model was fitted with the function “inla” in the INLA package for R^57^, with these settings:

- Input data was not aggregated; that is, one line in the dataset corresponded to a single trapping session.
- The expected density was defined by the argument E.
- Priors for the precision of model hyper-parameters (i.e., variance of the components of *u*), were provided by the BYM2 version of the estimation procedure^35^.

The success rate of boosted SIT was defined to identify traps with low mosquito density, with respect to their expected density *E*. We compared the fitted relative density ${\widehat{\theta }}$ with a predefined threshold $\theta ^{-}$. We used a common threshold for eggs and adults in each treated site: $\theta ^{-} = 0.20$. To estimate the success rate, 10,000 samples were drawn in the posterior marginals of the fitted relative density, and the proportion of simulated ${\widehat{\theta }}_{sim} \le \theta ^{-}$ was calculated. Boosted SIT was considered successful if $P({\widehat{\theta }}_{sim} \le \theta ^{-}) \ge 0.95$.

To compare the efficacy of non-boosted SIT vs. boosted SIT, we used egg data from La Vilavella (site 2) and its control site. La Vilavella (site 2) was exposed to non-boosted SIT in 2020 and 2022, and to boosted SIT in 2021.

1. The same oviposition traps were used to monitor mosquito eggs during the same months of exposition, throughout the 3 years. Therefore, we had a balanced design with *I* months of exposition and *J* traps, repeated three times, with non-boosted SIT in 2020 and 2022, and boosted SIT in 2021.
2. We computed the annual average density in 2020, 2021, and 2022, and then the differences in annual average density 2020–2021, 2022–2021, and 2022–2021 (Table 2). These original differences were the reference values.
3. Then, we randomly permuted the year in each block of month $i = 1, \ldots , I$ and trap $j = 1, \ldots , J$, and calculated the yearly grand mean on the permuted data set. We made 9999 random permutations.
4. Finally, we compared the original set of differences with these 9999 sets of permuted values.
5. All data and code are presented in this data paper: ^58^.

### Permits

Field trials involving the release of sterile males treated with pyriproxyfen were authorized.

- in Spain, on 16 July 2020, by the *Dirección General de Agricultura, Ganaderia y Pesca*, permit HYJHHQSA-UGILMZVJ-ATUPN7TE;
- in La Reunion, on 17 Feb. 2021, by the *Préfecture de Région de La Réunion*, *Bureau de l’Environnement*, *arrêté* 2021-282/SG/DCL, after an experimental authorization of biocide released by ANSES on 29 Dec. 2020, permit KF/SF 20-0351.

## Supplementary Information

Supplementary Information.

## Data Availability

The datasets used for the efficacy assessment, as well as the R code reproducing the analysis, are available in a data paper purposely written to accompany this article, which can be found at https://datadryad.org/stash/share/a7_X2OqcfPmE8QglPmGFvXiMO-AOP6kysXHqvi5j2mQ during the review process.
