# Supplementary material for: Suppression of Aedes mosquito populations with the boosted sterile insect technique in tropical and Mediterranean urban areas
Source: Sci Rep. 2025 May 21;15:17648. doi: 10.1038/s41598-025-02795-1 (PMC12095636; doi:10.1038/s41598-025-02795-1)
Supplement: Supplementary file 1 — Supplementary Information. [file 41598_2025_2795_MOESM1_ESM.pdf]

Supplementary information for

Suppression of *Aedes* mosquito populations with the boosted sterile insect technique in tropical and Mediterranean urban areas

J. Bouyer(*), D. Almenar Gil, I. Pia Mora, V. Dalmo Sorli, H. Maiga, W. Mamai, W. Mamai, I. Claudel, R. Brouazin, H. Yanado, L.-C. Gouagna, M. Rossignol, F. Chandre, M. Dupraz, F. Simard, T. Baldet, R. Lancelot(*)

*Corresponding author(s). E-mail(s): jeremy.bouyer@cirad.fr; renaud.lancelot@cirad.fr;

Supplementary Discussion

Monitoring tools

Monitoring total egg count is easy to implement. It requires much less time and manpower than monitoring adult mosquitoes, either emerging from nymphs, after hatching the eggs, and rearing larvae (as done in Spain), or sampled with BG-Sentinel traps (as conducted in La Reunion). Furthermore, Manica *et al.* (2017)[1] reported that total egg count provided reliable estimates of *Ae. albopictus* biting females. Though this indicator overlooks the biocide component of pyriproxyfen-boosted SIT, egg count is usually the main monitoring tool in large-scale field trials, or in routine mosquito-control programs based on SIT[2, 3]. For an assessment of the biocide component of boosted SIT, it should be associated with adult counts, at least on a sub-sample of egg catches, or by using adult traps.

Added value of spatial models

The reported space-time changes in the efficacy of boosted SIT should be understood as the product of several interactions. To begin with, the estimation of egg and adult density, based on recorded oviposition or BG-Sentinel trapping results, was influenced by trap settings, as well as factors related to the specific location of each trap, such as the abundance of artificial or natural containers, and micro-habitat characteristics. This was of special importance in La Reunion, where oviposition and BG-Sentinel traps were set to maximize the detection of *Ae. aegypti*, as well as its relative density with respect to *Ae. albopictus*[4, 5]. For instance, all the traps (oviposition and BG-Sentinel traps) were installed in the canopy of vacoa trees, because these trees were the only identified breeding site for *Ae. aegypti* in Saint-Joseph, where *Ae. albopictus* larvae were also present in equal densities.

Discernible zonal trends in density/success patterns (figs. 3-5) suggested potential associations with landscape factors. In the two Spanish treated sites, a noteworthy connection emerged, linking high mosquito densities, low treatment success, and proximity to areas characterized by tree cover and increased moisture (figs. 4-5). For instance, these sites were surrounded by citrus fields (fig. 1), where mosquito densities were higher than in urban environments[6]. These untreated areas were sources of re-invasion, a likely explanation of the spatial distribution of the CAR component of Poisson model random effect (SI figs. S9-S12). This might explain why the success rate was highest in the most central sectors of both treated areas (figs. 4-5), addressing both egg and adult stages. Distinct agricultural practices may influence mosquito densities in these neighboring zones. Irrigation methods (e.g., drip systems vs. flooding) and tree maintenance practices (including frequent pruning) could render agricultural habitats less suitable to *Ae. albopictus*. Conversely, the northwestern sector of La Vilavella where the success rate of boosted SIT was highest, was surrounded by arid Mediterranean shrub vegetation unsuitable to *Ae. albopictus*, and corresponded to areas where densities remained low (fig. 5 & SI figs S9-10).

The ability to highlight hot-spots and/or spatial trends in *Aedes* relative density depends on the study design. In particular, the sampling frame must account for this goal, while keeping the field and laboratory work in a sustainable range. In practice, a systematic, gridded sampling frame is often needed to detect spatial variations in relative density[7]. Such a sampling frame was adopted in this study, with an approximate resolution of one trap/ha, as a trade-off between bias and available manpower/time.

Validation of all phase-II milestones for boosted SIT

In terms of public health for vector-borne diseases, this experiment was the field component of a phase-II vector-control study, i.e., a small-scale efficacy assessment targeting the suppression of *Aedes* populations[8]. Besides the efficacy assessment, all the milestones for a validated phase-II mosquito-SIT[9] were fulfilled.

One of milestones of phase II is the identification of Complementary suppression methods [9]. Our trial showed that boosted SIT includes its own suppression component. Moreover, it may be combined to source reduction to account for larger breeding sites. Once the mosquito population is suppressed, SIT may be used to avoid the development of resistance to pyriproxyfen, and limit the contamination of the environment.

Permits were obtained for experimental use of pyriproxyfen for the field trials (see Methods). Although no pyriproxyfen formulation is presently (March 2024) authorized on the European-Union market to upscale the technique, the biocide is authorized until 2035, as well as several larvicidal formulations; the SIT component was authorized separately in La Reunion (see Methods).

In collaboration with the Saint-Joseph city hall (La Reunion), population was informed with press releases, as well as radio and TV broadcasts. All premises of the target and control areas were visited. Face-to-face interviews highlighted an excellent social acceptance of the technique: only one out of 176 visited households expressed concerns. In Spain, the trials were conducted in municipalities where SIT had been previously applied, and where communication campaigns had been carried out accordingly. The boosted SIT trial was supervised by the Agriculture and Environment Agency of the Valencia Regional Government. It was reported to the City Councils of cities from control and treated sites.

Production capacity of sterile males was established in Spain before this trial[6]. In La Reunion, all sterile male *Ae. aegypti* used in the trial were imported from the FAO-IAEA Insect Pest Control Laboratory (Vienna, Austria). In parallel, a weekly production capacity of 300,000 sterile male *Ae. albopictus* was established[10, 11].

Perspectives

Boosted SIT has two drawbacks: (i) it is not a green technology, and (ii) it might have non-target impacts because the effect of pyriproxyfen is not limited to *Aedes* mosquitoes. In La Reunion, we monitored the effect of pyriproxyfen on the emergence rate of chironomids observed in the same larval habitats than *Ae. aegypti*, as well as on the bee mortality rate in five sentinel bee haves in the control and treated sites. We did not observe any significant effect on both indicators (data not presented).

This study provides the proof of principle that pyriproxyfen-vectoring by sterile males offers an innovative way to transfer the biocide to females, and to larval habitats as well, in real environments[12]. In the next future, pyriproxyfen might be replaced with densoviruses which are specific to their insect hosts, and may even result in higher suppression allowed by their replication and high persistence in the infested breeding sites[13]. Their use would restore the specificity of SIT (lost with boosted SIT) while ensuring a greater boosting effect than pyriproxyfen[14, 15], with the potential of revolutionizing mosquito control.

Following this experiment, a wider-scale trial started in 2024 in La Reunion - in a 200-ha urban area, to assess the efficacy of pyriproxyfen-boosted SIT (i) to suppress both *Aedes* species and (ii) to mitigate the transmission of dengue virus (phase-III trial)[8]. It encompasses an entomological survey, as well as the monitoring of immunological, virological, and clinical indicators in humans. Its social acceptability will be formally assessed, as well as its possible environmental impacts.

Supplementary tables

SI tab. S1: Relative density, fitted with a spatial Poisson model, from data sampled from three zones treated with boosted SIT: Chemin Damour ($n = 7$ traps/collection day) in Saint-Joseph (site 1, La Reunion) from March to July 2021, La Vilavella (site 2, $n = 35$) and Polinyà de Xúquer (site 3, $n = 23$) in Valencia (Spain) from June to October 2021. The relative density was the density in traps from the treated zone, divided by the expected density, that is, the averaged density in traps from the control zone (site 1: Langevin, $n = 5$; site 2: Betxí, $n = 20$; site 3: Albalat de La Ribera, $n = 55$). The eggs were sampled with oviposition traps. In site 1, the adults were sampled with BG-Sentinel traps. In sites 2 and 3, they emerged from the eggs sampled with oviposition traps. In the column 'visualization', a vertical dashed line was drawn at relative density = 1, thus providing a bilateral test whether the 95% credible interval of relative density included 1, that is whether the suppression of the corresponding *Aedes* population was significant, with α = 1.

					95% credible limits		
treated zone	species	stage	exposure (month)	estimate	lower	Upper	visualization
Chemin Damour	*Aedes spp.*	eggs	1	1.32	1.16	1.50	
			2	0.46	0.40	0.52	
			3	0.24	0.16	0.34	
			4	0.77	0.66	0.89	
			5	0.67	0.54	0.82	
	Aedes aegypti	adults	1	0.34	0.28	0.43	
			2	0.60	0.44	0.80	
			3	0.33	0.23	0.46	
			4	0.19	0.11	0.30	
			5	0.09	0.06	0.15	
	Aedes albopictus	adults	1	0.89	0.65	1.18	
			2	1.11	0.82	1.46	
			3	0.51	0.34	0.73	
			4	0.30	0.14	0.57	
			5	0.41	0.27	0.59	
La Vilavella	*Aedes albopictus*	eggs	1	0.33	0.29	0.37	
			2	0.32	0.29	0.35	
			3	0.57	0.53	0.63	
			4	0.73	0.67	0.79	
	Aedes albopictus	adults	1	0.02	0.01	0.03	
			2	0.29	0.22	0.38	
			3	0.49	0.37	0.63	
			4	0.32	0.24	0.42	
Polinyà de Xúquer	*Aedes albopictus*	eggs	1	0.41	0.36	0.46	
			2	0.28	0.25	0.32	
			3	0.70	0.64	0.77	
			4	0.39	0.35	0.44	
	Aedes albopictus	adults	1	0.15	0.10	0.22	
			2	0.22	0.16	0.31	
			3	0.45	0.35	0.59	
			4	0.11	0.08	0.16	

Supplementary figures

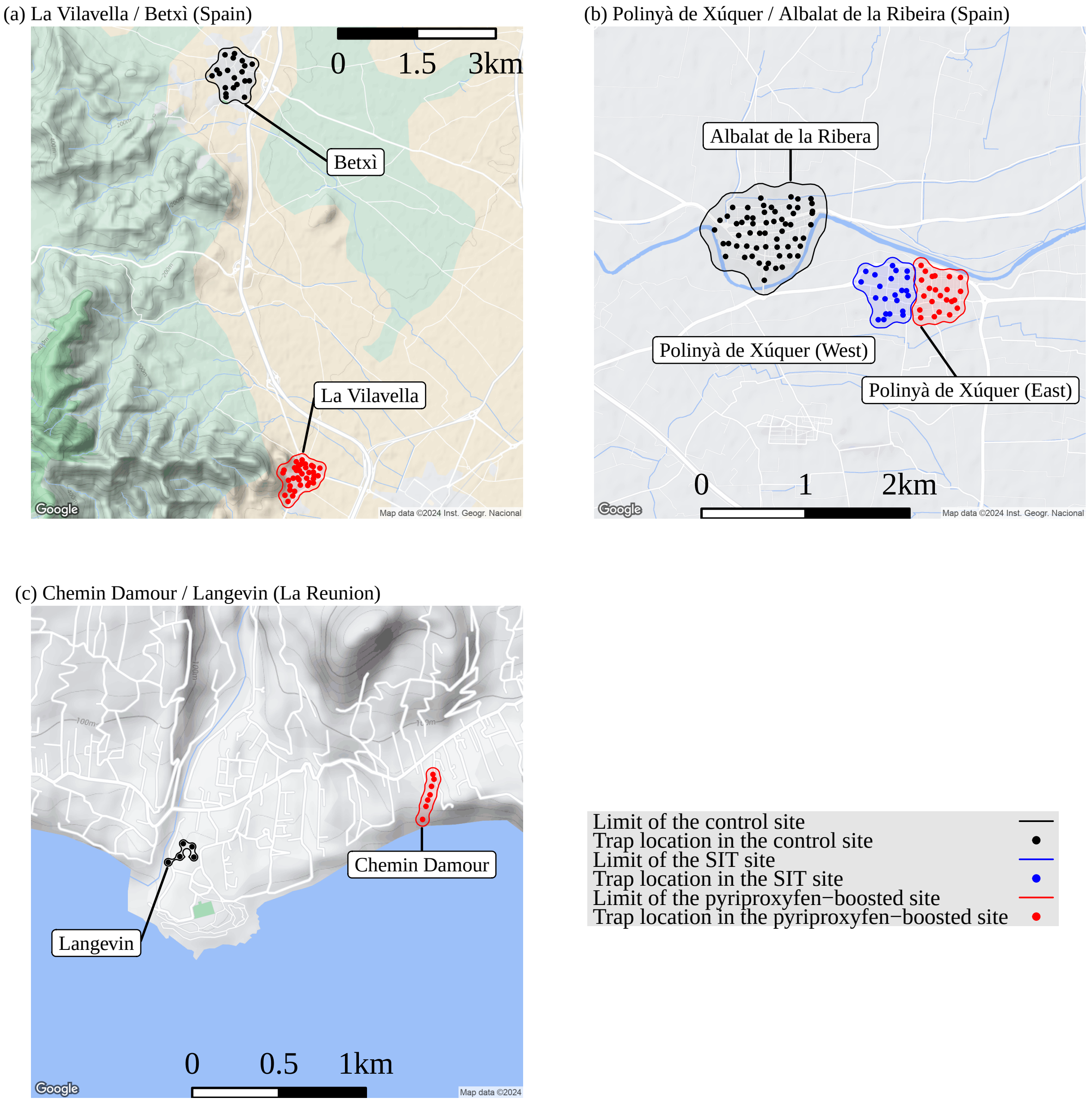

SI fig. S1: Location of control and treated sites during a boosted sterile insect technique field trial, implemented in three areas: two areas in the Region of Valencia (Spain: plots a and b), from June to October 2021, and one area in Saint-Joseph (La Reunion: plot c), from March to July 2021. The background maps in plots a, b and c were retrieved from the Google Maps Platform https://mapsplatform.google.com/, using functions available in the ggmap package for R https://github.com/features/packages version 4.0.0, together with a private API key. The administrative borders were retrieved from GADM https://gadm.org/data.html version 4.1.

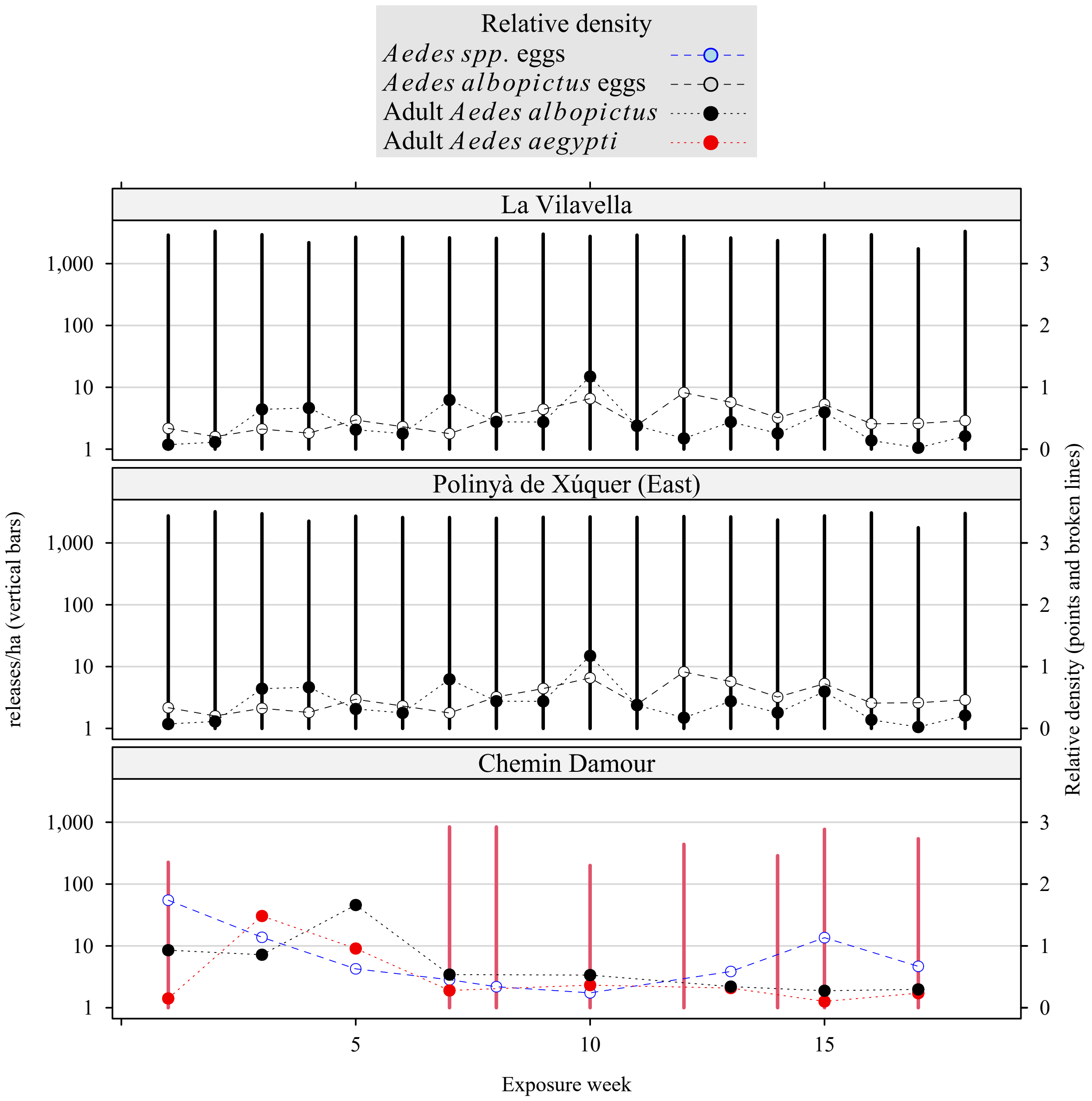

SI fig. S2: Weekly releases of pyriproxyfen-treated sterile, male *Aedes albopictus* (black, vertical bars) or *Aedes aegypti* (red, vertical bar during a boosted SIT field trial, from June to October 2021 in the Region of Valencia, Spain (La Vilavella and Polinyà de Xúquer), and from March to July 2021 in Saint-Joseph, La Reunion (Chemin Damour).

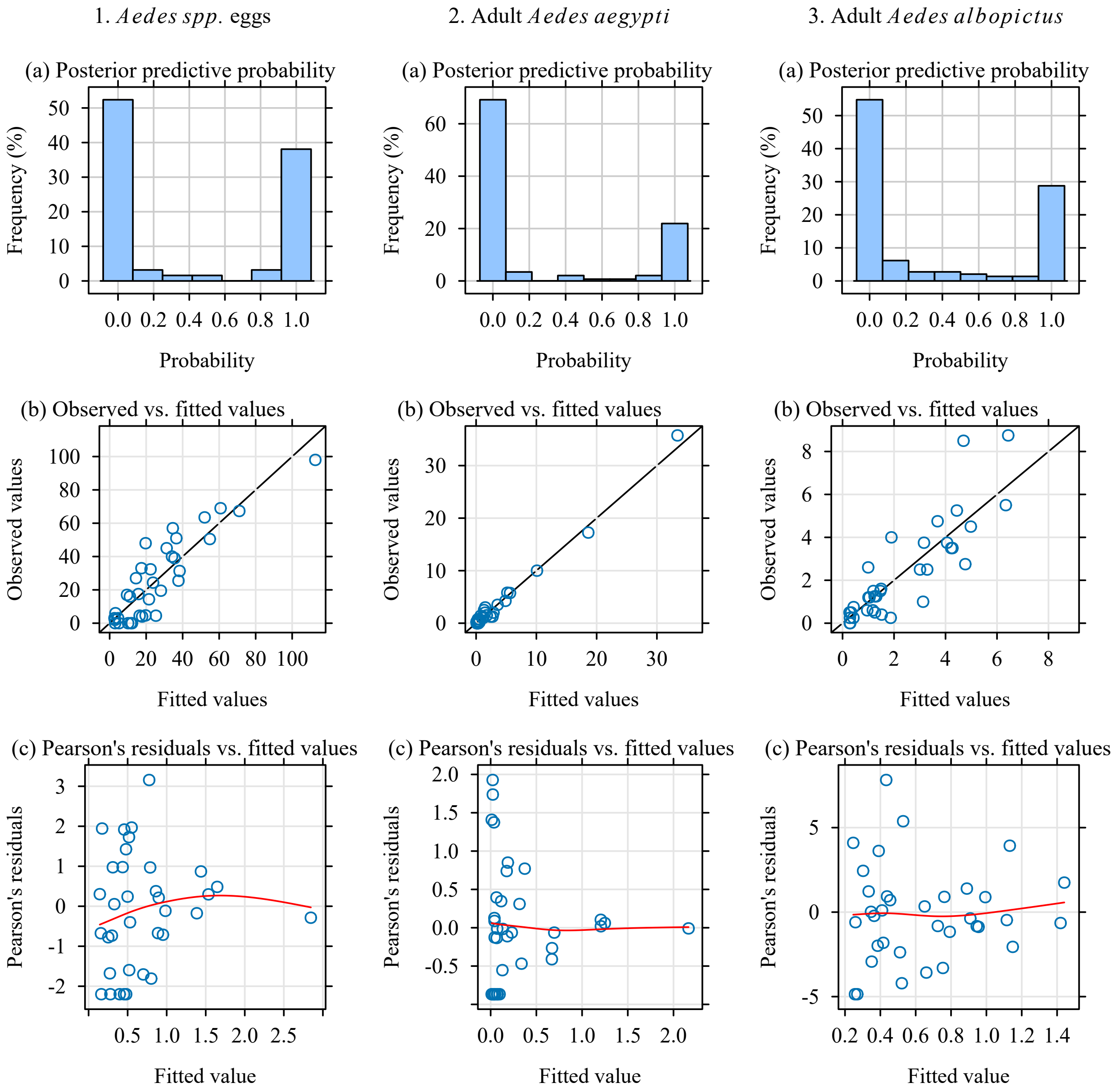

SI fig. S3: Goodness of fit for three spatial Poisson models of relative density of *Aedes* mosquitoes in Chemin Damour treated site (Saint-Joseph, La Reunion): 1. *Aedes spp.* eggs ($n = 7$ traps/collection day); 2. Adult *Aedes aegypti* ($n = 7$). 3. Adult *Aedes albopictus* ($n = 7$). Mosquitoes were sampled from March to July 2021. Eggs were sampled with oviposition traps. Adults were sampled with BG-Sentinel traps. The relative density was the observed density in traps located in the treated site, divided by the expected density, i.e., the averaged density in the Langevin control site ($n = 5$).

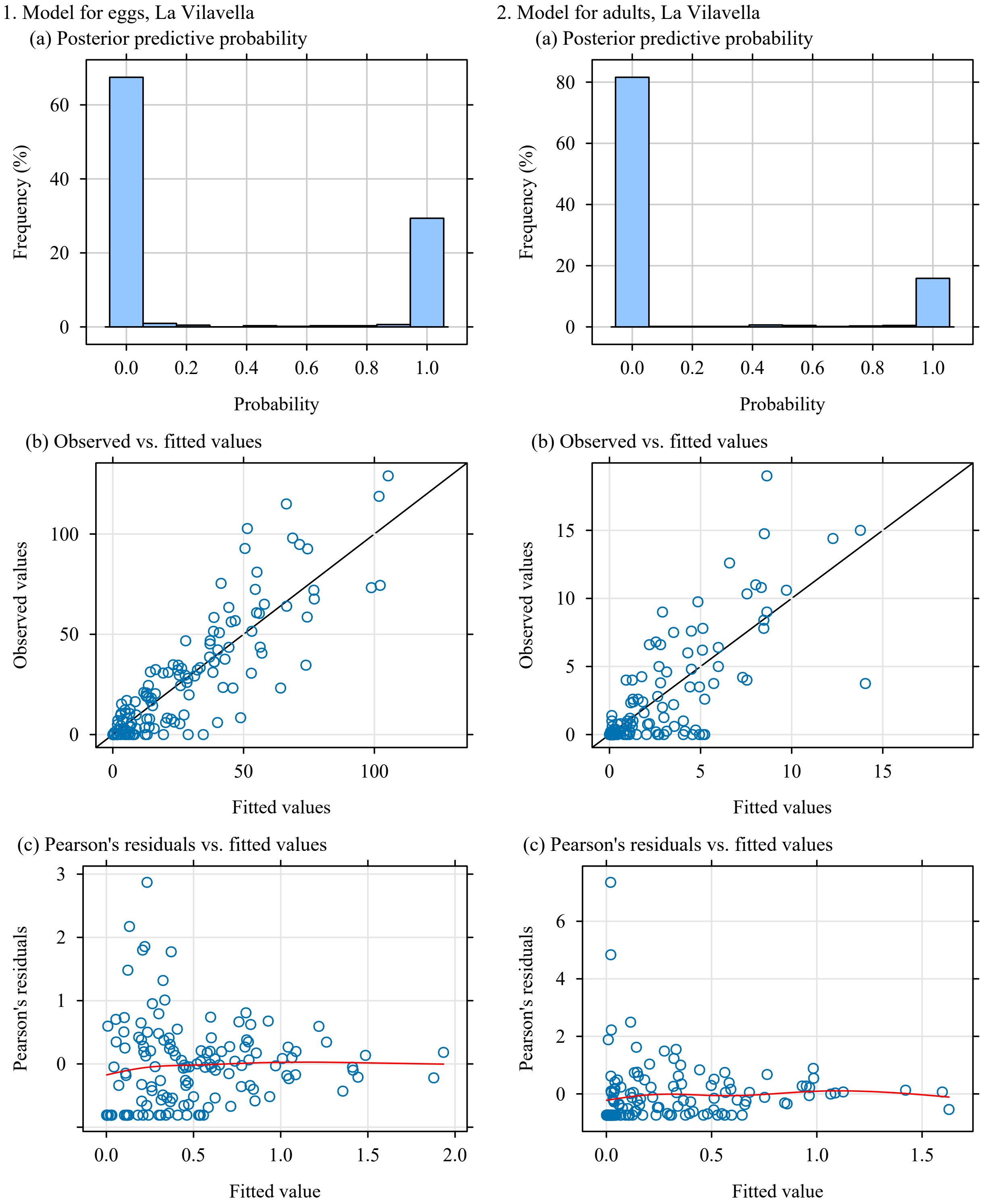

SI fig. S4: Goodness of fit for two spatial Poisson models of relative density of *Aedes albopictus* in La Vilavella (Valencia, Spain): 1. Eggs (n = 35 traps/collection day); 2. Adults (n = 35 traps). Mosquitoes were sampled from June to October 2021. Eggs were sampled with oviposition traps. Adults emerged for these eggs in the laboratory. The relative density was the observed density in traps located in the treated site, divided by the expected density, i.e., the averaged density in the Betxí control site (n = 20).

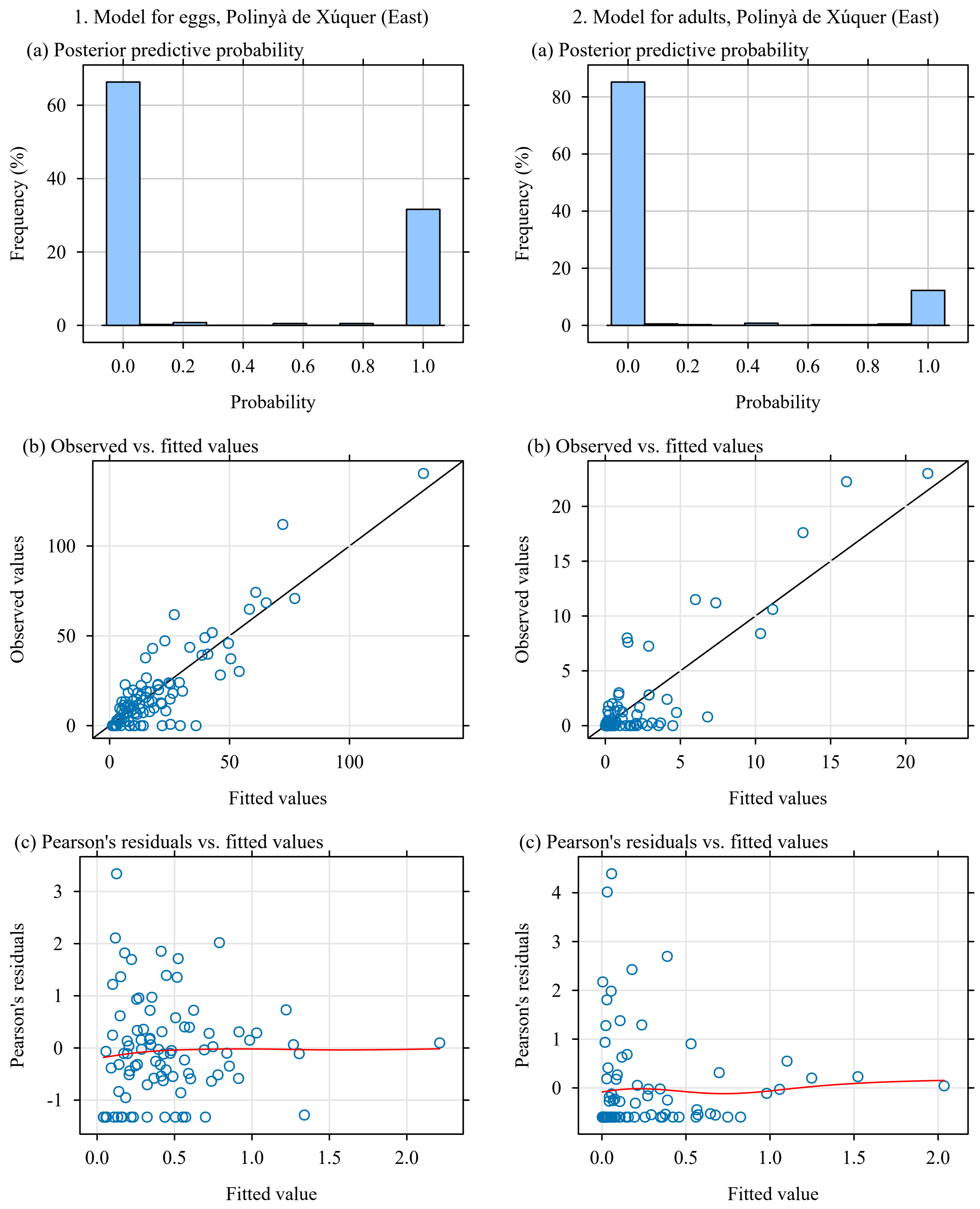

SI fig. S5: Goodness of fit for two spatial Poisson models of relative density of *Aedes albopictus* in Polinyà de Xúquer East (Valencia, Spain): 1. Eggs ($n = 23$ traps/collection day); 2. Adults ($n = 23$). Eggs were sampled with oviposition traps. Adults emerged for these eggs in the laboratory. The relative density was the observed density in traps located in the treated site, divided by the expected density, i.e., the averaged density in the Albalat de la Ribera control site ($n = 55$).

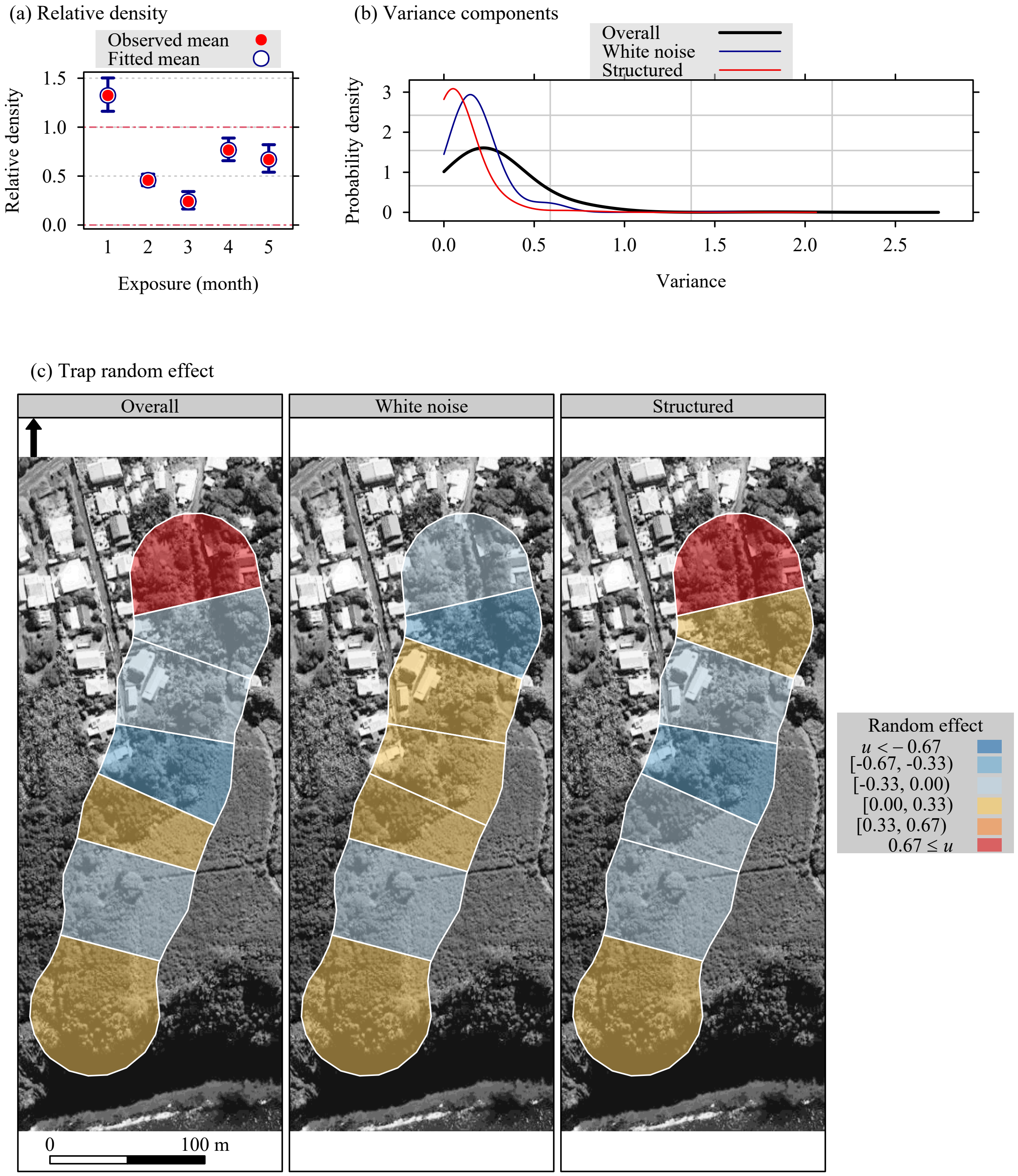

SI fig. S6: Main features of the spatial Poisson models of relative density of *Aedes* eggs in Chemin Damour (Saint-Joseph, La Reunion): (a) Fitted relative density and 95% credible interval; (b) Variance of the random effect components; (c) Spatial distribution of the random effect components. Mosquito eggs were sampled with oviposition traps from March to July 2021 ($n = 7$ traps/collection day). The relative density was the observed density in traps located in the treated site, divided by the expected density, i.e., the averaged density in the Langevin control site ($n = 5$). The background maps in plot c were retrieved from the Google Maps Platform https://mapsplatform.google.com/, using functions available in the ggmap package for R https://github.com/features/packages version 4.0.0, together with a private API key. The administrative borders were retrieved from GADM https://gadm.org/data.html version 4.1.

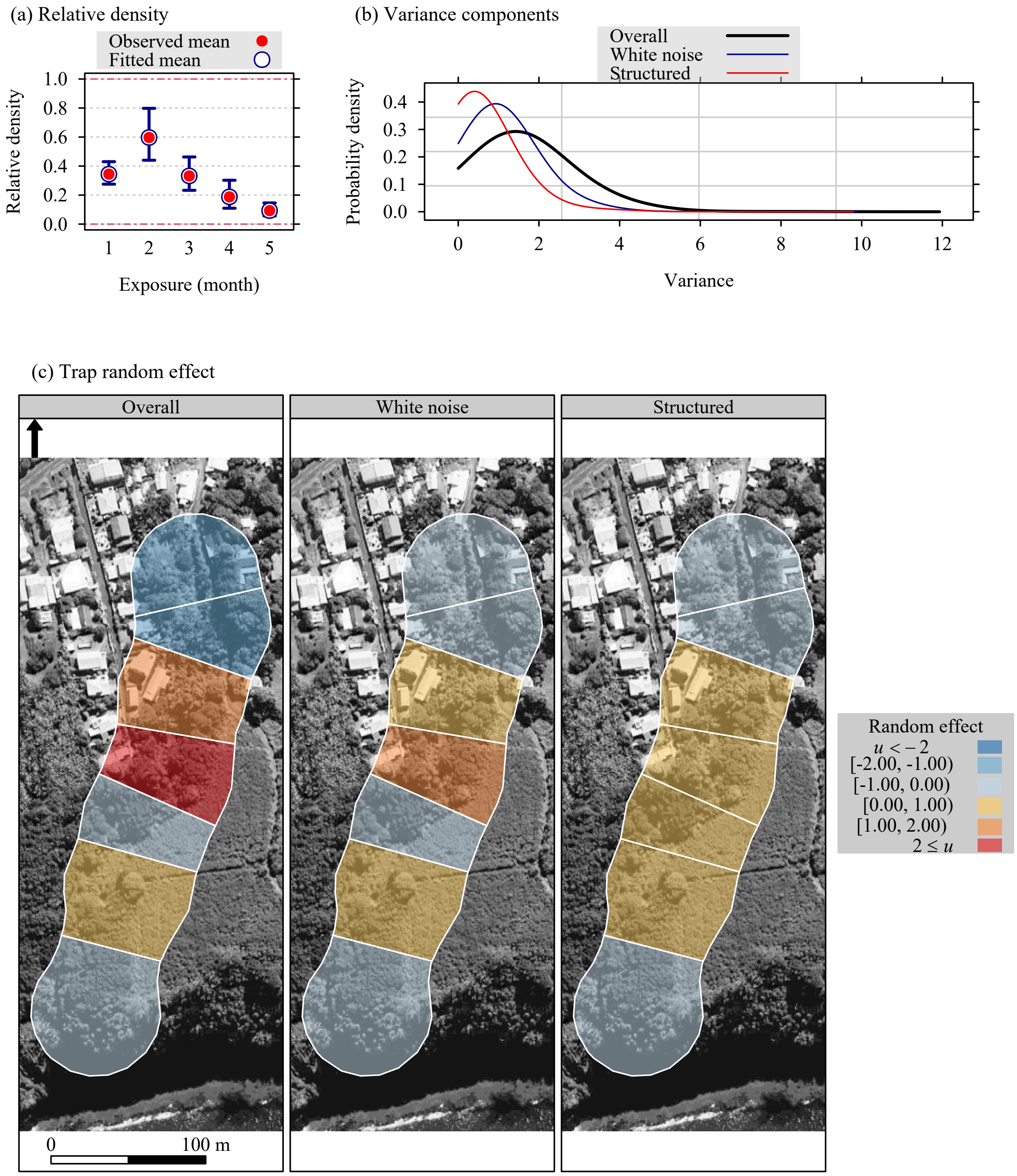

SI fig. S7: Main features of the spatial Poisson models of relative density of adult *Aedes aegypti* in Chemin Damour treated site (Saint-Joseph, La Reunion): (a) Fitted relative density and 95% credible interval; (b) Variance of the random effect components; (c) Spatial distribution of the random effect components. Adult mosquitoes were sampled with BG-Sentinel traps from March to July 2021 ($n = 7$ traps/collection day). The relative density was the observed density in traps located in the treated site, divided by the expected density, i.e., the averaged density in the Langevin control site ($n = 5$). The background maps in plot c were retrieved from the Google Maps Platform https://mapsplatform.google.com/, using functions available in the ggmap package for R https://github.com/features/packages version 4.0.0, together with a private API key. The administrative borders were retrieved from GADM https://gadm.org/data.html version 4.1.

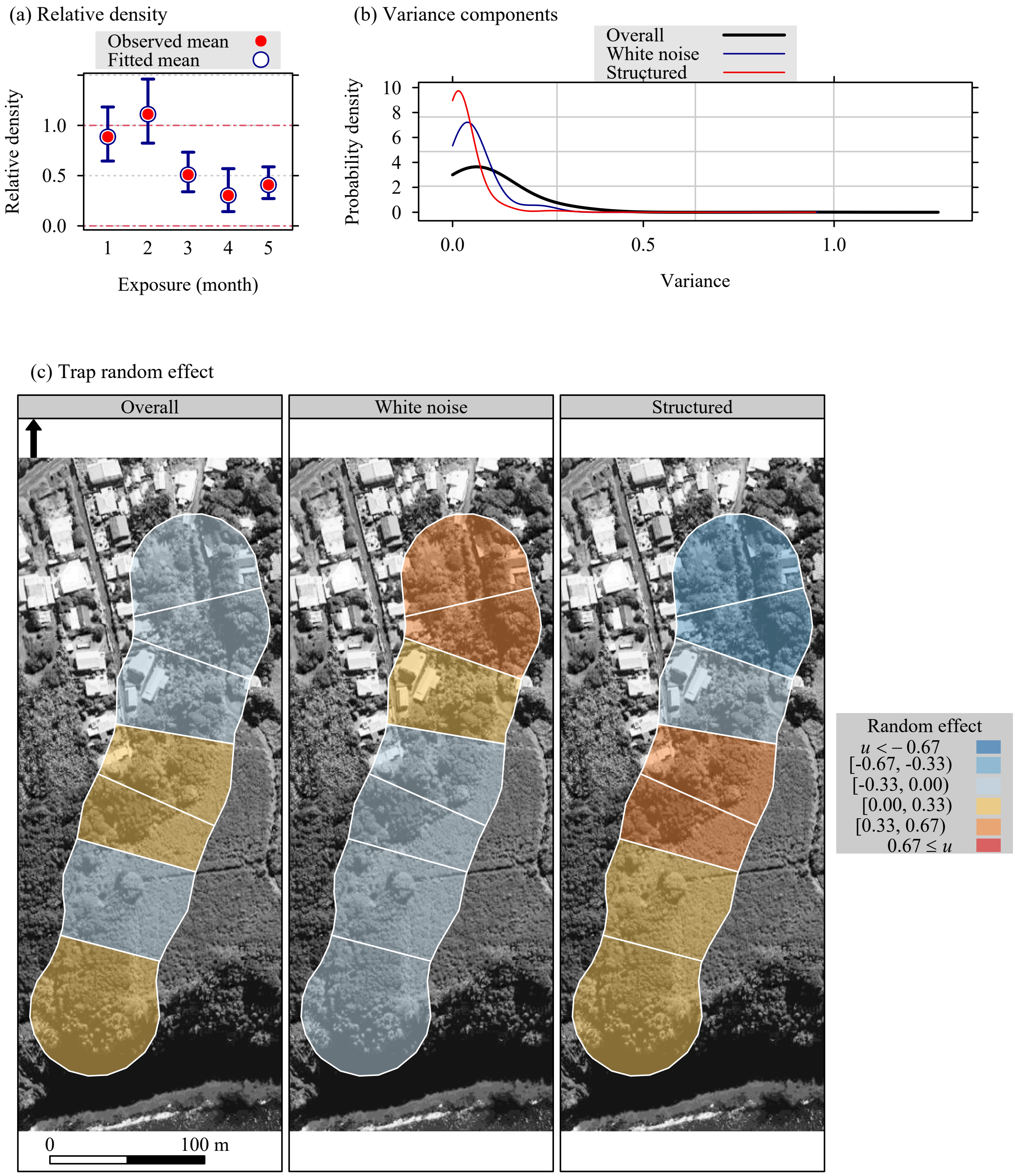

SI fig. S8: Main features of the spatial Poisson models of relative density of adult *Aedes albopictus* in Chemin Damour treated site (Saint-Joseph, La Reunion): (a) Fitted relative density and 95% credible interval; (b) Variance of the random effect components; (c) Spatial distribution of the random effect components. Adult mosquitoes were sampled with BG-Sentinel traps from March to July 2021 ($n = 7$ traps/collection day). The relative density was the observed density in traps located in the treated site, divided by the expected density, i.e., the averaged density in the Langevin control site ($n = 5$). The background maps in plot c were retrieved from the Google Maps Platform https://mapsplatform.google.com/, using functions available in the ggmap package for R https://github.com/features/packages version 4.0.0, together with a private API key. The administrative borders were retrieved from GADM https://gadm.org/data.html version 4.1.

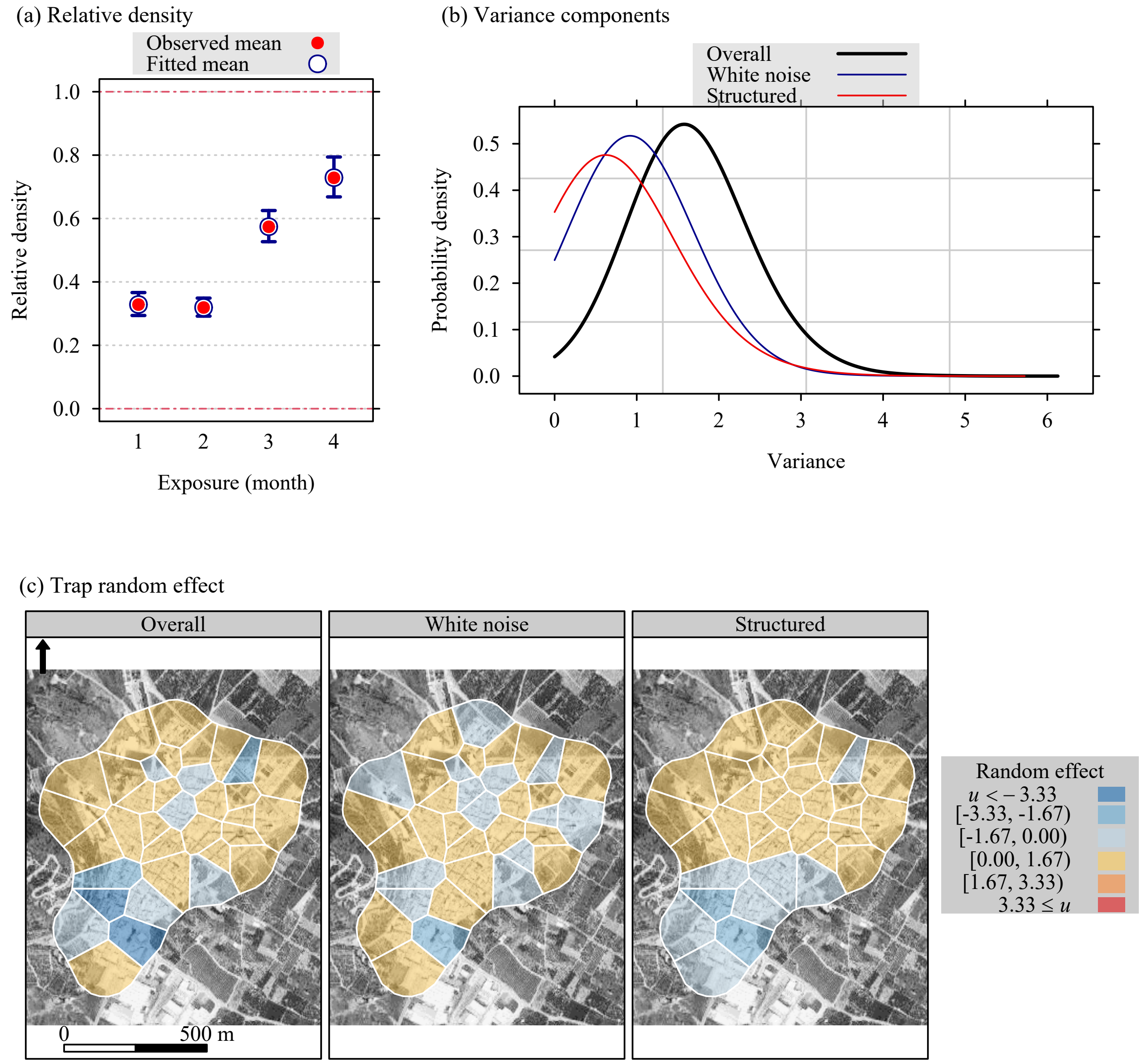

SI fig. S9: Main features of the spatial Poisson models of relative density of *Aedes albopictus* eggs in La Vilavella treated site (Valencia, Spain): (a) Fitted relative density and 95% credible interval; (b) Variance of the random effect components; (c) Spatial distribution of the random effect components. Eggs were sampled with oviposition traps from June to October 2021 ($n = 35$ traps/collection day). The relative density was the observed density in traps located in the treated site, divided by the expected density, i.e., the averaged density in the Betxí control site ($n = 20$). The background maps in plot c were retrieved from the Google Maps Platform https://mapsplatform.google.com/, using functions available in the ggmap package for R https://github.com/features/packages version 4.0.0, together with a private API key. The administrative borders were retrieved from GADM https://gadm.org/data.html version 4.1.

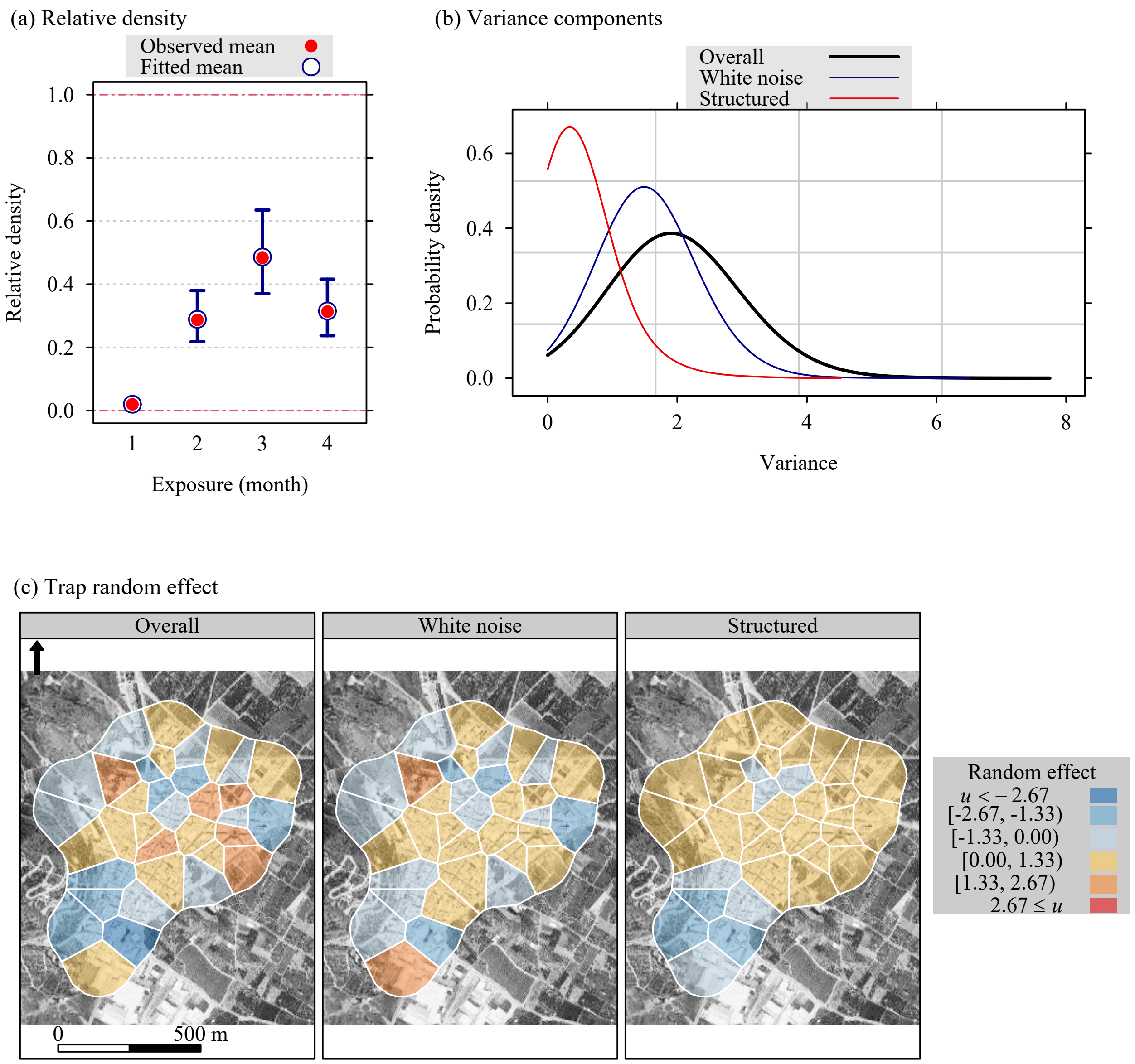

SI fig. S10: Main features of the spatial Poisson models of relative density of adult *Aedes albopictus* in La Vilavella treated site (Valencia, Spain): (a) Fitted relative density and 95% credible interval; (b) Variance of the random effect components; (c) Spatial distribution of the random effect components. Adult mosquitoes emerged from eggs sampled with oviposition traps from June to October 2021 ($n = 35$ traps/collection day). The relative density was the observed density in traps located in the treated site, divided by the expected density, i.e., the averaged density in the Betxí control site ($n = 20$). The background maps in plot c were retrieved from the Google Maps Platform https://mapsplatform.google.com/, using functions available in the ggmap package for R https://github.com/features/packages version 4.0.0, together with a private API key. The administrative borders were retrieved from GADM https://gadm.org/data.html version 4.1.

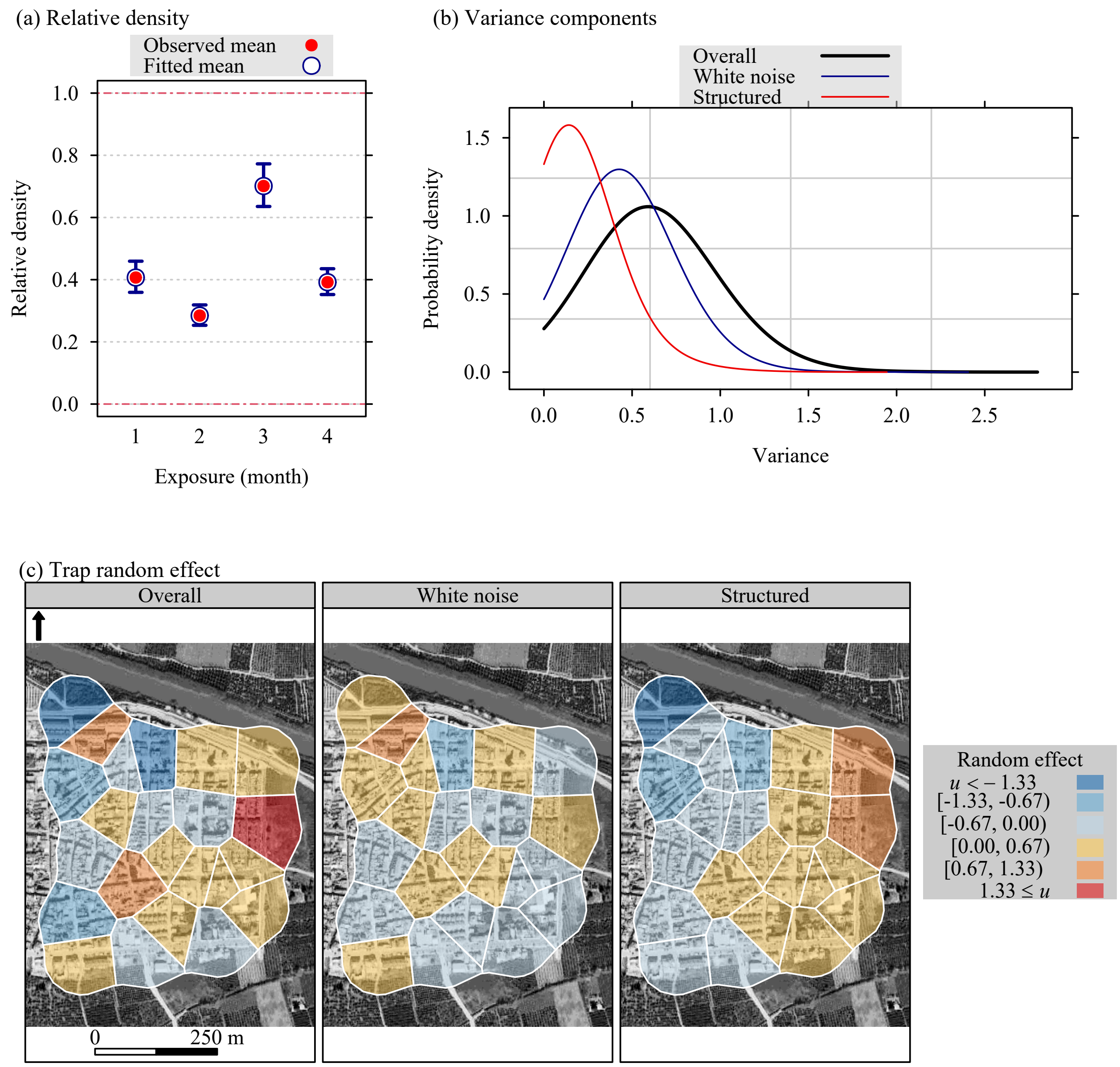

SI fig. S11: Main features of the spatial Poisson models of relative density of *Aedes albopictus* eggs in Polinyà de Xúquer treated site (Valencia, Spain): (a) Fitted relative density and 95% credible interval; (b) Variance of the random effect components; (c) Spatial distribution of the random effect components. Mosquito eggs were sampled with oviposition traps from June to October 2021 ($n = 23$ traps/collection day). The relative density was the observed density in traps located in the treated site, divided by the expected density, i.e., the averaged density in the Albalat de la Ribera control site ($n = 55$). The background maps in plot c were retrieved from the Google Maps Platform https://mapsplatform.google.com/, using functions available in the ggmap package for R https://github.com/features/packages version 4.0.0, together with a private API key. The administrative borders were retrieved from GADM https://gadm.org/data.html version 4.1.

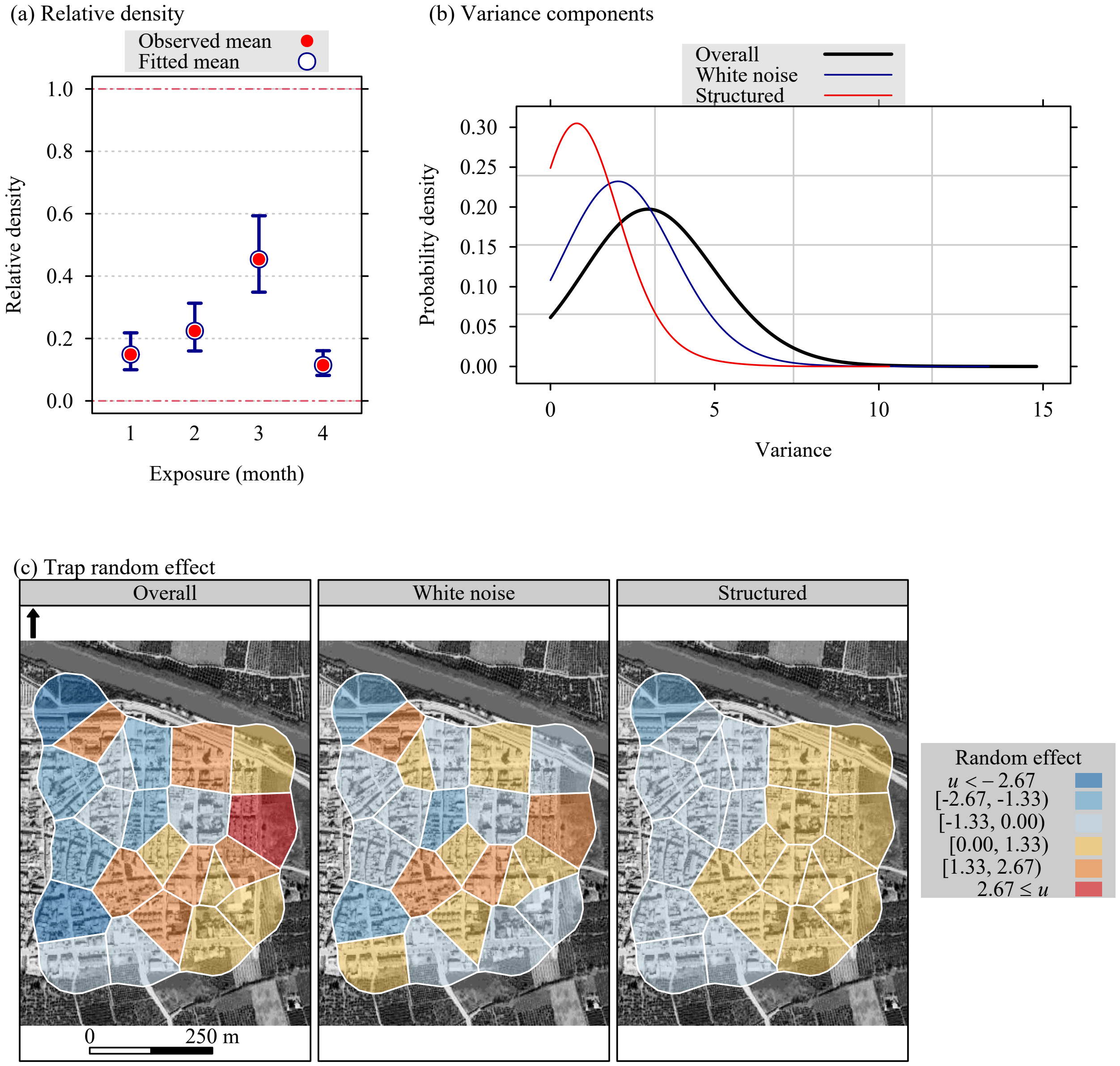

SI fig. S12: Main features of the spatial Poisson models of relative density of adult *Aedes albopictus* in Polinyà de Xúquer treated site (Valencia, Spain): (a) Fitted relative density and 95% credible interval; (b) Variance of the random effect components; (c) Spatial distribution of the random effect components. Adult mosquitoes emerged from eggs sampled with oviposition traps and reared in the laboratory from June to October 2021 ($n = 23$ traps/collection day). The relative density was the observed density in traps located in the treated site, divided by the expected density, i.e., the averaged density in the Albalat de la Ribera control site ($n = 55$). The background maps in plot c were retrieved from the Google Maps Platform https://mapsplatform.google.com/, using functions available in the ggmap package for R https://github.com/features/packages version 4.0.0, together with a private API key. The administrative borders were retrieved from GADM https://gadm.org/data.html version 4.1.

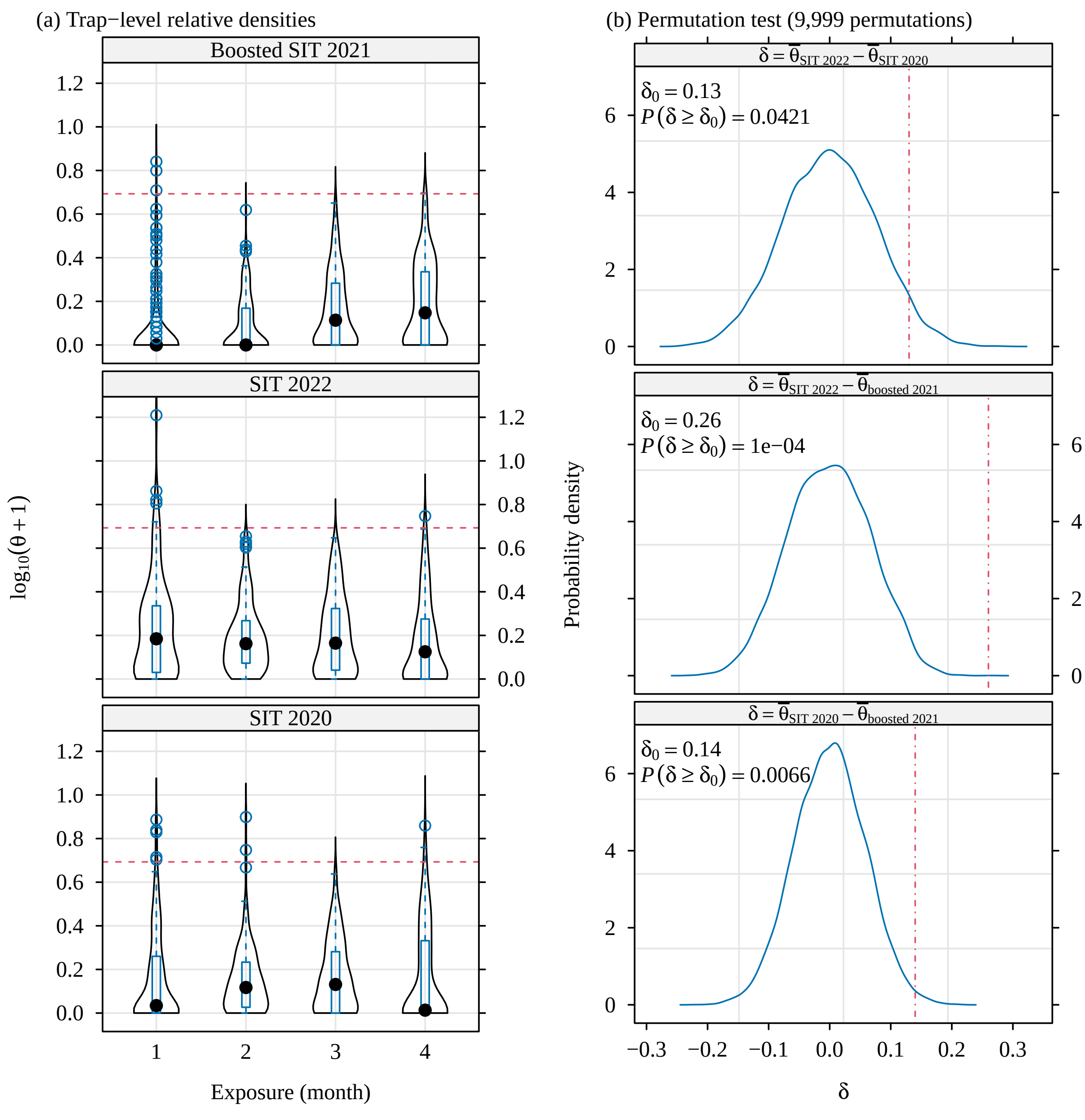

SI fig. S13: Relative density of *Aedes albopictus* eggs in mosquito populations treated either with standard, or boosted SIT in La Vilavella (Valencia, Spain). (a) Trap-level density (θ) - the dashed, red horizontal line was drawn at $\theta = 1$ and (b) Permutation test of the difference δ_o in averaged density (9,999 permutations). SIT was implemented in 2020 and 2022, and boosted SIT in 2021. The observed density was standardized with corresponding data (same year) from the Betxí control site.

References

[1] M. Manica, R. Rosà, A. della Torre, B. Caputo, From eggs to bites: do ovitrap data provide reliable estimates of Aedes albopictus biting females? PeerJ **5**, e2998 (2017). https://doi.org/10.7717/peerj.2998

[2] R. Bellini, M. Carrieri, F. Balestrino, A. Puggioli, M. Malfacini, J. Bouyer, Field Competitiveness of Aedes albopictus (Diptera: Culicidae) Irradiated Males in Pilot Sterile Insect Technique Trials in Northern Italy. J. Med. Entomol. **58**(2), 807–813 (2020). https://doi.org/10.1093/jme/tjaa235

[3] R. Gato, Z. Menéndez, E. Prieto, R. Argilés, M. Rodríguez, W. Baldoquín, Y. Hernández, D. Pérez, J. Anaya, I. Fuentes, C. Lorenzo, K. González, Y. Campo, J. Bouyer, Sterile Insect Technique: Successful Suppression of an Aedes aegypti Field Population in Cuba. Insects **12**(5), 469 (2021). https://doi.org/10.3390/insects12050469

[4] R. Brouazin, I. Claudel, R. Lancelot, G. Dupuy, L.C. Gouagna, M. Dupraz, T. Baldet, J. Bouyer, Optimization of oviposition trap settings to monitor populations of Aedes mosquitoes, vectors of arboviruses in La Reunion. Sci. Rep. **12**, 18450 (2022). https://doi.org/10.1038/s41598-022-23137-5

[5] I. Claudel, R. Brouazin, R. Lancelot, L.C. Gouagna, M. Dupraz, T. Baldet, J. Bouyer, Optimization of adult mosquito trap settings to monitor populations of Aedes and Culex mosquitoes, vectors of arboviruses in La Reunion. Sci. Rep. **12**, 19544 (2022). https://doi.org/10.1038/s41598-022-24191-9

[6] C. Tur, D. Almenar, M. Zacarés, S. Benlloch-Navarro, I. Pla, V. Dalmau, Suppression Trial through an Integrated Vector Management of Aedes albopictus (Skuse) Based on the Sterile Insect Technique in a Non-Isolated Area in Spain. Insects **14**(8), 688 (2023). https://doi.org/10.3390/insects14080688. URL https://www.mdpi.com/2075-4450/14/8/688

[7] L. Sedda, B.M. Taylor, A.E. Eiras, J.T. Marques, R.J. Dillon, Using the intrinsic growth rate of the mosquito population improves spatio-temporal dengue risk estimation. Acta Trop. **208**, 105519 (2020). https://doi.org/10.1016/j.actatropica.2020.105519. URL https://www.sciencedirect.com/science/article/pii/S0001706X20301704?via=ihub

[8] WHO, How to design vector control efficacy trials: guidance on phase III vector control field trial design provided by the Vector Control Advisory Group. Tech. rep., World Health Organization (2017). 7. Licence: CC BY-NC-SA 3.0 IGO

[9] J. Bouyer, H. Yamada, R. Pereira, K. Bourtzis, M.J.B. Vreysen, Phased Conditional Approach for Mosquito Management Using Sterile Insect Technique. Trends Parasitol. **36**(4), 325–336 (2020). https://doi.org/10.1016/j.pt.2020.01.004

[10] L. Marquereau, B. Derepas, A. Leclercq, L.C. Gouagna, *Standardization of irradiation process of Aedes albopictus males under massrearing conditions in support of SIT program in La Reunion Island*, in *Mosquito Irradiation, Sterilization and Quality Control. Report of the First Research Coordination Meeting of an FAO/IAEA Coordinated Research Project, held in Vienna, Austria, from May 31 to June 4, 2021 (Virtual)*, ed. by IAEA (Vienna, Austria, 2021). URL https://www.iaea.org/sites/default/files/22/06/d44004-report_1strcm_red.pdf

[11] L. Marquereau, H. Yamada, D. Damiens, A. Leclercq, B. Derepas, C. Brengues, B.W. Dain, Q. Lejarre, M. Proudhon, J. Bouyer, L.C. Gouagna, Upscaling irradiation protocols of Aedes albopictus pupae within an SIT Programme in Reunion Island. Sci. Rep. **2024** (2024). https://doi.org/10.21203/rs.3.rs-3829903/v1

[12] P.G. Kevan, L. Shipp, G. Smagghe, Ecological intensification: Managing biocomplexity and biodiversity in agriculture through pollinators, pollination and deploying biocontrol agents against crop and pollinator diseases, pests and parasitess. Entomovectoring for Precision Biocontrol and Enhanced Pollination of Crops **2020**, 19–51 (2020)

[13] R.M. Johnson, J.L. Rasgon, Densonucleosis viruses (densoviruses) for mosquito and pathogen control. Curr. Opin. Insect Sci. **28**, 90–97 (2018). https://doi.org/10.1016/j.cois.2018.05.009

[14] J. Bouyer, F. Chandre, J. Gilles, T. Baldet, Alternative vector control methods to manage the Zika virus outbreak: more haste, less speed. The Lancet Global Health **4**(6), e364 (2016). https://doi.org/10.1016/S2214-109X(16)00082-6

[15] A. Perrin, A.S. Gosselin-Grenet, M. Rossignol, C. Ginibre, B. Scheid, C. Lagneau, F. Chandre, T. Baldet, M. Ogliastro, J. Bouyer, Variation in the susceptibility of urban Aedes mosquitoes infected with a densovirus. Sci Rep. **10**(1) (2020). https://doi.org/10.1038/s41598-020-75765-4
